# Language-based affect assessments capture experiment-induced changes beyond rating scales

**DOI:** 10.3758/s13428-026-03200-z

**Published:** 2026-11-03

**Authors:** Oskar Bång, Zhuojun Gu, August H. Nilsson, Veerle C. Eijsbroek, Clara Wiebel, Adithya V. Ganesan, Katarina Kjell, H. Andrew Schwartz, Oscar N. E. Kjell

**Affiliations:** 1 Department of Psychology, Lund University, Lund, Sweden; 2 Oslo Business School, Oslo Metropolitan University, Oslo, Norway; 3 Department of Computer Science, Stony Brook University, Stony Brook, NY USA; 4 Department of Computer Science, Vanderbilt University, Nashville, TN USA

**Keywords:** Language-based assessments, Natural language processing, PANAS, Mood induction procedure

## Abstract

Language-based assessments (LBAs) have shown strong convergent and criterion validity cross-sectionally and longitudinally. However, no study has tested whether LBAs of affect can capture experiment-induced changes (i.e., *causal validity*). In this randomized mixed-design experiment, participants (*N =* 892) completed a *mood induction procedure* comprising different conditions (*church, shopping mall,* and *park*) using physical locations (*N* = 153) and online video clips (*N* = 739). Participants reported their pre- and post-induction affect through open-ended responses and ratings (i.e., the Positive and Negative Affect Schedule; PANAS). Using large language models and machine learning, we compared how accurately PANAS and language responses classified the experimental conditions. The LBA derived from the language responses exhibited significantly higher accuracy compared to the PANAS scores in classifying the conditions in both the training set (cross-validated AUC = .74 vs. .63), the online holdout set (AUC = .76 vs. .70), and the offline holdout set (AUC = .67 vs. .53). Apart from showing higher predictive accuracy, we assessed LBAs of specific affective dimensions (valence, arousal, and basic emotions). These analyses revealed that positive valence was most strongly expressed in the park condition, while anger was particularly elevated in the mall condition. We also visualized language differences across conditions, showing more calm and peaceful expressions in the park condition and more tired and bored expressions in the mall condition. These findings support the causal validity of language-based affect assessments and their ability to provide insights into affective states. We discuss the potential of using LBA in experimental psychological research.

**Supplementary Information:**

The online version contains supplementary material available at https://doi.org/10.3758/s13428-026-03200-z.

## Introduction

When people are asked about their emotional state in a natural setting (e.g., *How are you feeling?*), they typically respond with descriptive language (e.g., *I'm feeling pretty good*) rather than expressing themselves in numerical values or closed-ended categories (e.g., *7* *= Completely Agree*; Kjell et al., 2019). Language is a natural and powerful tool for conveying one’s state of mind (Pennebaker et al., 2003). How individuals use words to express themselves and their emotional states provides a valuable source of information for understanding them (Boyd & Schwartz, 2021; Pennebaker, 2011). However, psychological constructs such as affect are often measured using predefined, closed-ended numerical rating scales (Flake et al., 2017). The conventional rating-scale method of assessing subjective states has advanced psychological science in the past decades. They are easy to use, fast, and mostly interpretable. However, they have relatively limited dimensionality (measuring only a few predefined items), range (fixed scale range, e.g., 1–7), and resolution (inability to capture nuances between predefined items and fixed ranges) of the response information as compared to free expressions (Kjell et al., 2024), which often fail to capture transient, moment-to-moment fluctuations in everyday emotional experiences (Sun et al., 2019).

Recent advances in artificial intelligence have improved the ability to measure and quantify linguistic behavior, offering the possibility of numerically capturing emotional states and traits derived from open-ended language in reproducible language models (Althoff et al., 2016; Min et al., 2021; Srivastava et al., 2022). These assessments, referred to as language-based assessments (LBAs), measure mental states or traits by transforming language into numeric representations (*word embeddings*) used to create assessment scores. In this work, we focus specifically on *probed* LBAs, which rely on open-ended responses to targeted questions (e.g., Kjell et al., 2019), rather than on unstructured language from sources such as social media (e.g., Schwartz et al., 2013).

Probed LBAs have demonstrated validity by showing strong convergence with corresponding rating scales, i.e., convergent validity (e.g., Gu et al., 2024; Kjell et al., 2022; Nilsson et al., 2024b), and by cross-sectionally and longitudinally predicting theoretically related outcomes more accurately than corresponding ratings, i.e., criterion validity (Gu et al., 2024; Kjell et al., 2021a, b, c; Nilsson et al., 2024a). In addition to convergent and criterion validity, causal validity—the ability to capture change resulting from a change in the to-be-measured construct (Borsboom et al., 2004)—is essential for measurement validation (as further described below). To our knowledge, no research has directly compared the ability of LBAs and rating scales to detect experimentally induced changes in affect.

To address this gap, the present study evaluates the performance of probed LBAs and rating scales in a randomized experimental setting. We assess the ability of these methods to classify participants’ randomly assigned mood induction procedure conditions (church, shopping mall, or park) based on affect descriptions and ratings collected before and after the induction. The randomly assigned condition serves as a ground truth criterion to test whether each method can detect systematic affective changes elicited by the induction. We also show how LBAs can characterize the nature of these changes, using theoretically grounded pretrained models to assess dimensions such as valence, arousal, and basic emotions, along with visualizations of statistically significant words and topics that differentiate conditions.

### Conceptualizing affect and emotions

*Affect* and *emotions* are complex, related but distinct psychological phenomena theorized through multiple frameworks. Dimensional models describe affect in terms of continuous dimensions such as the *Circumplex Model of Affect*, which arranges affective states in a two-dimensional space defined by valence (pleasant-unpleasant) and arousal (activation-deactivation; Russell, 1980a, b). In contrast, discrete emotion theories emphasize basic, evolutionarily rooted emotions such as anger, fear, or joy, understood as qualitatively distinct categories (Ekman, 1992). Constructivist models argue that emotional experience is assembled from core affect, conceptual knowledge, and social context (Barrett, 2017). These perspectives underscore the complexity of understanding and measuring affective change. Hence, a comprehensive assessment of affect should account for both the underlying dimensions and the discrete labels individuals use to describe their states. Recognizing these complexities, the present study is conceptually grounded in psychological constructionist models (Barrett, 2017). This framework suggests that language is not merely a post hoc report of a state but an active component in constructing emotional meaning (Hoemann et al., 2025). Affective states are inherently situated, requiring individuals to flexibly adapt their expressions to changing environmental demands and dynamic social contexts (Aldao, 2013; Greenaway et al., 2018). Consequently, we adopt theoretically informed, pretrained LBA models designed to quantify affect from language in terms of dimensional valence and arousal, as well as discrete emotions, while acknowledging that such operationalizations necessarily simplify the rich, socially situated nature of emotional language. Transforming open-ended emotional language into numeric embeddings offers a method for quantifying affect while maintaining its inherent complexity, integrating dimensional, discrete, and constructivist affect perspectives (Hoemann et al., 2024).

### Language-based assessments

LBAs can be seen as a broad collection of methods that measure psychological constructs by transforming natural language into numerical representations (e.g., counts or word embeddings), language summaries (e.g., topics), or other language features. The term LBA dates back to 2015, when topics of Facebook status updates were used to assess self-reported personality traits (Park et al., 2015). In the current study, we focus mainly on large language model (LLM)-based methods using probed responses as the language source. Probed LBAs rely on open-ended responses to targeted questions (e.g., “How do you feel right now?”), which allows researchers to capture specific momentary states. Compared to unstructured LBAs based on passively collected or naturally occurring language (e.g., from social media or medical records), probed LBAs allow for more standardized input and experimental control and typically align the language source in time and content with the construct of interest (Gu et al., 2024; Kjell et al., 2024). Recent developments in large language models (LLMs) have driven significant progress in natural language processing (Bommasani, 2021; Devlin et al., 2018; Min et al., 2021; Vaswani et al., 2017). Research suggests that LLM-based LBAs have favorable psychometric properties (Kjell et al., 2024). For example, probed LBAs of depression, life satisfaction, and harmony in life have converged with corresponding rating scales at *r* =.81,.80, and.85 (Gu et al., 2024; Kjell et al., 2022). Such high levels of convergence approach the theoretical upper limit, since internal reliability sets an upper limit on convergence (Muchinsky, 1996; Spearman, 1961). However, validating LBA against rating scales is an important but limited benchmark since scales do not represent the objective truth of these constructs (Cronbach & Meehl, 1955; Eijsbroek et al., 2025a, b; Krosnick, 1999). Consequently, these studies may overlook effects that rating scales cannot capture.

To validate LBA beyond rating scales, studies have compared it with actual behaviors and other external criteria. For example, researchers have validated unstructured LBAs against external criteria involving demographics and behaviors ranging from daily sleep hours to Facebook behaviors (Eichstaedt et al., 2018; Schwartz et al., 2014; Son et al., 2023). For behaviors, probed LBAs of anxiety and depression have tended to predict sick leave and health care visits more strongly than the corresponding ratings (Gu et al., 2024); probed LBA of ecological momentary assessment-based well-being has predicted alcohol consumption nearly four times more accurately than the corresponding ratings (Nilsson et al., 2024a); and probed LBA of harmony in life has predicted theoretically relevant cooperative behavior from a give-some dilemma game up to four times more accurately than the corresponding ratings (Kjell et al., 2021a, b, c). Thus, LBA not only reaches the theoretical upper limit of accuracy in converging with corresponding ratings, but often surpasses them in criterion validity—especially when predicting behaviors and outcomes.

### Causal validity

Causal validity is the ability of a measure to be causally influenced by the targeted construct (Borsboom et al., 2004). Critically, establishing causal validity does not require, and is often hindered by, comparison to a criterion measure. Instead of relying on correlations between measures, which can be misleading if the criterion itself is noisy, the focus is on the causal change in measurement (e.g., experimental manipulation). Showing how a construct affects test-item responses can help distinguish valid measures and their sensitivity. For example, a valid affect measure should be affected by a valid mood induction procedure. In turn, one should observe differences in affect measurement scores between those randomly assigned to different mood induction conditions and, consequently, be able to distinguish the different conditions. While several LBAs have shown convergent validity by predicting the corresponding rating scales (Gu et al., 2024; Kjell et al., 2022; Sametoğlu et al., 2024) as well as criterion validity by predicting theoretically related outcomes both cross-sectionally (Kjell et al., 2021a, b, c; Nilsson et al., 2024b; Schwartz et al., 2014) and longitudinally (Eichstaedt et al., 2018; Son et al., 2023), these forms of validity rely on correlational evidence. This is problematic because correlation alone does not demonstrate measurement validity; for instance, age is highly correlated with height, but age is not a measure of height. Convergent and criterion validity cannot, on their own, establish that an assessment truly measures affect. To address this, we ask whether LBAs, compared to ratings, can capture changes in affect following an experimental manipulation, thereby demonstrating causal validity.

To test the causal validity of LBAs and ratings, this study used a mood induction procedure (Merz et al., 2013) by randomly assigning participants to different locations (church, mall, park). Mood induction procedures are widely used to elicit emotional states (e.g., happiness, sadness, anxiety) and examine their effects on cognitive, emotional, and behavioral processes (Kučera & Haviger, 2012). By intentionally altering mood in a controlled way, mood induction procedures provide a strong test of whether different measures can detect and differentiate these changes, helping to establish a causal link between mood and measured outcomes (Marcusson-Clavertz et al., 2019). This study focuses on short-term effects, with meta-analyses showing mood induction procedures typically produce about a one-standard-deviation change in immediate mood compared to controls (Joseph et al., 2020; Westermann et al., 1996).

### The dimensionality of measures

A measure's dimensionality or a model’s dimensionality may affect its ability to capture change when testing causal validity. Dimensionality refers to the number of components or predictors in a measure or model—such as factors in factor analysis, items in a rating scale, or predictors in a regression model. In psychology, when exploring and validating different models and measures of personality (e.g., the Big Five-factor model; Costa & McCrae, 1992), item-level measures have been shown to explain more and generate more accurate predictions than factor-level models (Nielsen & Kajonius, 2024; Seeboth & Mõttus, 2018; Stewart et al., 2022; Vainre et al., 2025), which implies that measures with a higher dimensionality can hold higher predictive power.

In this study, we compare different levels of dimensionality across measures. Using the Positive and Negative Affect Schedule (PANAS) rating scale (Watson et al., 1988), we assess predictive accuracy using either composite scores (lower dimensionality) or individual items (higher dimensionality). For LBAs, we examine both high-dimensional word embeddings (typically 768 or more dimensions) and theoretically grounded, lower-dimensional outputs that capture core affective dimensions such as valence, arousal, and basic emotions. By systematically comparing these levels of dimensionality, we aim to identify which representations best capture participants’ affective states across experimental conditions while also offering theoretical insight.

### Language visualizations

Beyond measuring outcomes, LBA can offer deeper insights into how people express mental states by visualizing significant language differences across different traits, states, or behaviors. For example, analyses of social media language have revealed differences in personality and health risks (Schwartz et al., 2013; Eichstaedt et al., 2015). Similarly, affect descriptions can show meaningful distinctions—such as words reflecting socializing and exercise (e.g., *friends*, *running*) being linked to higher well-being, while words indicating imbalance (e.g., *too*, *much*) relate to lower well-being (Nilsson et al., 2022). Even when rating scales correlate highly, language can reveal distinct profiles, such as harmony in life emphasizing *balance* and *peaceful*, versus satisfaction with life highlighting *fulfilled* and *happy* (Kjell et al., 2019). These visualizations can help to better understand individuals’ affective states across different experimental conditions and changes over time.

### Present study

We evaluate the ability of probed LBAs versus PANAS to capture affective change. Participants’ language responses were used to directly classify experimental conditions (i.e., data-driven classifications) as part of our pre-registered analyses. We hypothesized that LBAs would outperform rating scales in classifying participants’ conditions: specifically, that a model combining affect description embeddings and PANAS scores would outperform PANAS alone (H_1_), and that affect description embeddings alone would also outperform PANAS (H_2_). To better understand the nature of affective differences across conditions and explain what drives the data-driven classifications, we conducted further analyses using theoretically grounded assessments of affective dimensions (valence, arousal, and basic emotions) and language-based visualizations through differential language analysis.

## Method

### Participants

Participants were recruited from Malmö city (Sweden) for the *offline sample* and from various countries on Prolific for the *online sample* (Table 1). Participants were restricted to English fluency and using PCs with Google Chrome browsers. Participants who scored positively for symptoms of major depression were screened out in the offline sample (*N* = 106, 69.3%), and they were automatically screened out at Prolific in the online sample recruitment before they entered our survey; hence, no data were collected on them. The offline sample comprised 157 participants recruited from July 2022 to May 2023. We excluded four participants with incomplete answers. From the remaining sample (*N =* 153), 127 were collected during 2022 before the pre-registration, and 26 were collected during 2023 after the pre-registration^1^. We collected the online sample between April and May 2024. We asked participants who did not watch the entire video or fast-forward it to resubmit (*N* = 39). We collected all online participants after they submitted the pre-registration. Table 1 provides study sample information and participant demographics. All participants in the offline sample were from Sweden. The countries of residence in the online sample included nine countries: Australia, Canada, Ireland, New Zealand, South Africa, Sweden, the UK, and the US. Participants who scored positively for symptoms of major depression were screened out in the offline sample (*N* = 106, 69.3%), and they were automatically screened out at Prolific in the online sample recruitment before they entered our survey and hence no data were collected on them.

**Table 1:** Study sample information and participant demographics

Sample	Offline	Online
*N* total	153	739
*N* across conditions Church/Mall/Park	45/54/54	254/248/237
Mean age in years (SD; range)	31.6 (12.09; 18–75)	42.8 (13.77; 18–89)
Gender Female/Male/Other	77/72/4	424/308/7
Survey and original response language	Swedish	English
Median completion time in mins (SD)	17.30 (8.04)	15.61 (8.88)
Religion Non-religious/Christian/Non-Christian religious/Preferred not to say	105/29/6/13	390/264/72/13

### Instruments^2^

***Screening*** The *Patient Health Questionnaire* PHQ-2 (Löwe et al., 2005) was used to screen out participants. It comprises two items (i.e., *“Over the last 2 weeks, how often have you been bothered by any of the following problems? 1. Little interest or pleasure in doing things. 2. Feeling down, depressed, or hopeless”*) answered with rating scales ranging from “1 = not at all” to “4 = nearly every day”. Participants with a score of 3 or above were screened out.

#### The language-based affect question

To assess participants’ current affective state, we used an open-ended question adapted from Kjell et al. (2019):

*“How do you feel right now? Describe the feelings that you experience right now. Please answer the question by writing 5 descriptive phrases that describe your feelings right now. Write 1 to 10 words for each phrase.”*

The open-ended question was translated into Swedish for the offline sample; all the original questions are available in the pre-registration material.

#### The affect rating scale

*Positive and Negative Affect Schedule* (PANAS; Watson et al., 1988) is a widely used rating scale designed to measure affect in diverse contexts (Merz et al., 2013); it measures affect using 20 items that relate to various emotional states. Participants were asked to indicate how much they feel each emotional state using a rating scale ranging from “0 = not at all” to “4 = very much”. Half of the items relate to the Positive Affect Scale (e.g., *inspired*), and the other half capture the Negative Affect Scale (e.g., *nervous*). Research supports the psychometric utility of the PANAS across different populations and languages, employing various time course instructions to tap into trait-level (e.g., last year, past month) and state-level (e.g., past day, today) affect (Cotigă, 2012; Merz et al., 2013; Terracciano et al., 2003; von Humboldt et al., 2017; Watson & Clark, 1999; Watson et al., 1988). We used the Swedish version of the scale from Bergman & Daukantatie (2009) for the offline sample. Both the online and offline samples showed favorable psychometric properties (see Supplementary Material Table S1).

#### Intervention

The mood induction procedure involved asking participants to “take in” and be affected by three different environments/locations in Malmö: inside a commercial shopping mall (*Triangeln*), inside a church (*St Johannes Church*), or a park (*Magistratsparken* offline and *Pildammsparken* online). The instructions for the offline sample were:

“*First, we want you to sit comfortably.*

*Take a few deep breaths to land here and now.*

*See where you are and “take in” the surroundings.*

*Listen to the sounds around you. Examine whether you can smell any scents.*

*See what’s around you.*

*Take the time you need and try to stay in what you are experiencing for a few minutes. When you feel ready, move on by pressing "Next"*.

In the video instructions, participants were instructed not to fast-forward and to read the instructions carefully:

*Now, we would like you to watch a video showing a place – we would like you to imagine that you are there. See if you can let your mood be influenced by what you see in the video.*

*Here's how to make the most of this "journey":*

*Begin by positioning yourself comfortably in your seat. Make any adjustments needed to ensure you can relax fully without distractions.*

*Take a few deep breaths to land here and now. Allow yourself to become fully present in this moment.*

*When the video starts, see where you are and take in the surroundings in the video. Notice the colors, the shapes, and the movements.*

*Pay attention to the sounds in the video.*

*When imagining yourself being in the place shown in the video: How does it feel? What emotions does it evoke?*

*When you are ready, press "Next" to start the video.*

All videos were 201 seconds long, and the experiment ensured that the video was automatically downloaded in mp4 format (480p) before the participant could start the study.

### Procedure

This study obtained ethical approval from the Swedish Ethical Review Authority (Dnr 2021–06693-01). Potential participants were approached to partake in a study regarding how the physical environment affects one's emotions. To be eligible, participants had to be at least 18 years old. Upon providing consent, participants were screened for depression using the PHQ-2 questionnaire. Only individuals who scored two or below, indicating non-depression, were allowed to proceed with the study.

**Fig. 1:**
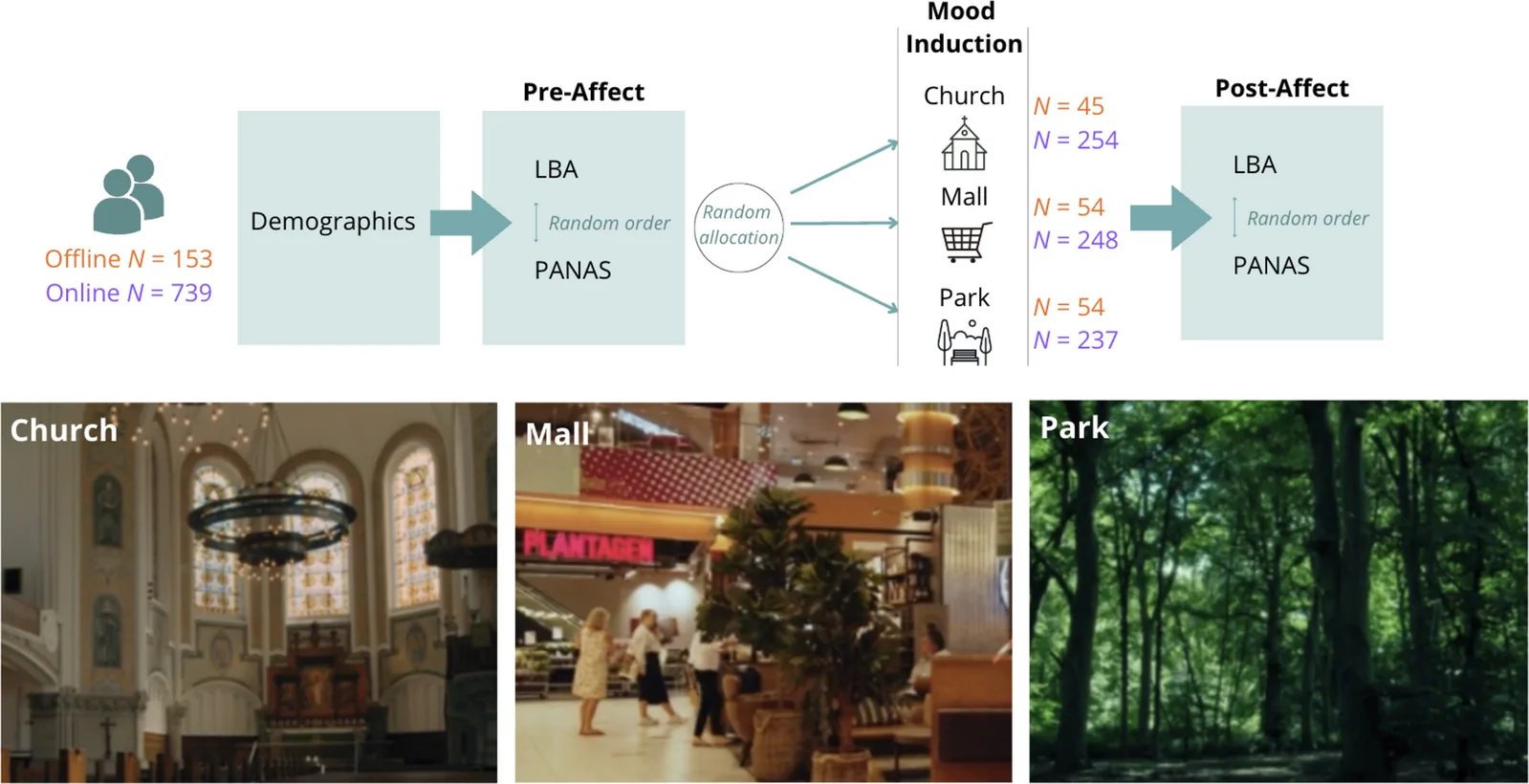
Overview of the experiment procedure. *Note.* *LBA* = language-based assessment, *PANAS* = Positive and Negative Affect Schedule

The procedure (see Fig. 1) began with participants answering demographic questions and other personality measures not used in this study. We randomized the order of presentation for these measures. Next, participants completed the PANAS, the open affect question, and provided a voice recording of their current affective state. The order of these was also randomized.

Following these initial assessments, participants were randomly assigned to one of three conditions: visiting a nearby church, shopping mall, or park. Upon arrival at their assigned location, participants recorded a standardized question before receiving the mood induction procedure. After completing the mood induction procedure at each location, participants answered the same affective state questions and rating scales as before the intervention, in randomized order. The process concluded with participants recording a standardized question outside each location.

Finally, participants were debriefed regarding the aims of the study. Data collection after the pre-registration phase followed the same procedure as before, except the park condition was excluded due to winter weather conditions.

### Conditions

The condition locations were chosen based on 1) convenience; all three places were within a short physical distance for the offline conditions, 2) distinctiveness; all conditions were distinct from each other, and 3) impact; the different locations should theoretically impact and elicit differences in affect. For example, in association with the mall, spending money on things and oneself is linked to lower levels of happiness compared to prosocial spending (Dunn et al., 2008, 2014; Kumar et al., 2020). For the park, connecting and interacting with nature is associated with higher levels of well-being and lower levels of stress (Bratman et al., 2012). In relation to the church environment, everyday spiritual experiences have been shown to increase positive affect directly (Whitehead & Bergeman, 2012).

### Pre-registration

The data collection and analyses were pre-registered. In the main analyses, we made one extension by including a more recent LLM (the results from the preregistered model are presented in Supplemental Material 1) and excluded one condition (Market) to harmonize the online and offline samples (analyses including the Market condition are presented in the Supplemental Material 1). The offline sample was pre-registered as part of a prior Master’s thesis study^3^.

### Analytical method

The analysis compared the ability of different measures to capture changes in affect. We tested this by examining whether LBAs or rating scales could more accurately classify which experimental condition participants were exposed to during the mood induction procedure. We used one dataset for training (online; 586 participants) and two different holdout sets for evaluation (online and offline; 153 participants each). For text analyses, training sizes have been shown to reach high convergent accuracy after 100 participants, with slight to diminishing increases in accuracy after 300 participants and upwards (Gu et al., 2024). These numbers informed the sample size. We divided the online training data using reproducible randomness, stratified for gender, age, and study condition. We first evaluated different combinations of PANAS and LBA of affect to classify conditions using multinomial logistic regression. We systematically evaluated the role of dimensionality (e.g., is PANAS capturing more relevant information as a composite score or as individual items?). We then evaluated theoretically grounded assessments of valence, arousal, and basic emotions and, finally, visualized the language related to the different conditions and compared the language related to the online and offline data.

#### Condition-related word removal

According to the pre-registration, direct references to the experimental conditions (i.e., “Mall,” “Church,” and “Park”) were excluded from the post-induction responses used in the classification analyses (although they were retained in the visualizations for interpretative purposes). To further mitigate potential contextual leakage, we implemented additional, more stringent filtering procedures, as detailed in the Supplemental Material 1. These included three approaches: i) automated identification of context-sensitive terms using ChatGPT-4o; ii) augmentation of the automated list through manual review to ensure comprehensive exclusion of context-revealing words; and iii) application of the above filtering steps followed by restriction of the text to only affect-related terms drawn from the Linguistic Inquiry and Word Count (LIWC), including the positive emotion and negative emotion dictionaries (Boyd et al., 2022).

#### Manipulation check

We evaluated whether the experiment worked as intended by examining whether there was any change in emotion between pre- and post-induction assessment of affect. We tested whether there was a significant difference in rated affect (from PANAS) by conducting a 2 × 3 mixed ANOVA (pre- post intervention scores + Condition + pre- and post scores × Condition). We also performed a manipulation check of change in affect based on the predicted valence (pre- and post-intervention) of the language responses.

#### Evaluating affect sensitivity of PANAS and LBAs

##### Evaluating PANAS

To assess its ability to capture changes in affect, we systematically evaluated PANAS’s ability to classify experimental conditions using three methods. The first method combined PANAS scores, combining pre- and post-induction affect scores in four ways. This included using i) post-induction PANAS, ii) the difference between pre- and post-induction PANAS, iii) the concatenation of pre- and post-induction PANAS, and iv) the concatenation of pre-, post-, and the difference between pre- and post-induction PANAS. Previously, the concatenation (i.e., combination/addition of embedding vectors across the dimensional space, such that two vectors of, for example, 768 dimensions turn into one vector of 1536 dimensions) of pre- and post-measures and their difference scores has shown the highest accuracy in predicting mood changes (Ganesan et al., 2022). The second method examined the breadth of emotions, meaning we compared only positive emotion items, only negative emotion items, and both positive and negative emotion items. The third method examined PANAS dimensionality: we tested concatenating PANAS into composite positive and negative emotion scores and using these as predictors, but we also tested individual PANAS items as predictors, resulting in many more predictors and dimensions than using only the composite scores. Combining these three methods produced 28 models, which we evaluated on cross-validated training data and online and offline holdout data (See supplementary materials S4 for an overview of models). All models classified the study conditions by ridge multinomial logistic regression (see section Predictive models).

##### Evaluating language-based assessments

We systematically evaluated the language data using two methods. The first method matched the PANAS approach, combining the pre- and post-induction measures in four ways. This included using i) post-induction language, ii) the difference between post- and pre-intervention language, iii) the concatenation of pre- and post-induction language, and iv) the concatenation of pre- and post-induction language and their difference (difference embeddings were achieved through vector subtraction, where the numerical representation of the pre-induction response was subtracted from the post-induction response). Second, we compared two LLMs, including the pre-registered RoBERTa Large (Liu et al., 2019) and the newer Mixed Bread Large (Li & Li, 2023). RoBERTa Large has previously demonstrated strong performance among LLMs in social science tasks (Choi et al., 2023; Ganesan et al., 2021; Ziems et al., 2023). However, the newer Mixed Bread Large has shown promising performance (as of March 2024, the model from Li & Li (2023)) ranked among the top of the Massive Text Embedding Benchmark (MTEB) list shown in Enevoldsen et al. (2025). Combining these two methods resulted in eight LBA models. We performed ridge multinomial logistic regressions to classify the study conditions as described above. The predictor variables were the numerical representations of the language (word embeddings).

#### Natural language processing

##### Word embeddings

We transformed the language responses into word embeddings, vectors that numerically represent the language's latent meaning. Each language input is represented as a vector in a high-dimensional space across several layers, with the vector’s position reflecting its meaning relative to other words in the corpus (Boyd & Schwartz, 2021). Each layer represents a slightly different contextual meaning. We transform these into word embeddings using large pre-trained language models: RoBERTa Large and Mixed Bread Large (layer 23) were used.

##### Language translation

For the offline holdout data, which was in Swedish, we used the DeepL API and the *deeplr* R-package (Zumbach & Bauer, 2021) to translate the affect phrases of the offline sample from Swedish to English. A psychology PhD student, independent of this study and blind to the study objectives and procedures, checked and corrected the translations. The student’s native language was Swedish.

#### Predictive models

##### Statistical modeling

Word embeddings can be trained to make LBA models that, in turn, can predict an outcome, such as experiment conditions or semantic characteristics (e.g., the valence of a text; Kjell et al., 2023). To achieve this, the dimensions of the word embeddings were used as the predictors in ridge regression models to classify the experiment conditions. Ridge regression penalizes the coefficients to avoid overfitting (Hoerl & Kennard, 1970). We used a penalty range from 10⁻⁶ to 10⁶, increasing by a factor of 10 at each step (i.e., 10⁻⁶, 10⁻^5^, …, 10⁶). To evaluate models with different penalties in the training set, we employed nested tenfold cross-validation, where the data was repeatedly divided into ten sets, including nine training sets and one holdout test set. We then randomly split each of the nine training sets into a development set (75% of the data) and a test set (the remaining 25%) to evaluate the developed models. We used the best-performing penalty in the final model (for further explanation, see Kjell et al., 2023). We used multinomial logistic ridge regression to test the classification accuracy of the 28 PANAS-based and eight LBA models, described in the “Evaluating PANAS” and the “Evaluating LBAs” sections above. We also concatenated the predictors of the best-performing PANAS-based and LBA models and tested if this increased the accuracy.

##### Model performance

We evaluated model performance on the online and offline holdout sets by computing AUC values (area under the ROC curve). This metric measures the overall accuracy of a logistic regression based on the sensitivity (i.e., true-positive rate) and specificity (i.e., true-negative rate). A model with 100% sensitivity and 100% specificity has an AUC of 1. A model with no classification ability has an AUC of.50. For the multinomial models, we used multinomial AUC as evaluation, which is the average one-vs.-all AUC of each pairwise comparison. To test whether one model performed significantly more accurately at categorizing the conditions than another, we calculated the difference between resampled AUC scores from model 1 and model 2, then tested whether this difference was significantly different from zero.

#### Theoretically grounded language-based assessments

To assess theoretically grounded dimensions of affect and emotions, we created and used pre-trained LBA models to assess valence (i.e., pleasant/unpleasant), arousal (i.e., calm/exciting), and basic emotions (i.e., anger, disgust, fear, joy, sadness, and surprise) of the participant's responses. We evaluated the theoretically grounded LBAs using multinomial ridge regression, as explained above in the predictive models section. All pre-trained LBA models are based on Mixed Bread layer 23 embeddings (Li & Li, 2023).

##### Language assessed for affect dimensions

The valence and arousal LBA models were pre-trained using a dataset including words with valence and arousal annotations (i.e., ratings on a scale from 1 to 9; Warriner et al., 2013). Valence was rated based on the stimulus pleasantness, and arousal based on stimulus intensity. Each annotator rated the words solely on one of the two dimensions. There were 1827 annotators, and all words had at least 15 ratings (87% had 18 to 30 annotations (ratings) per word). The models are trained on the average ratings of each word. The pre-trained valence model exhibited a tenfold cross-validated Pearson’s *r* =.83, and the pre-trained arousal model exhibited a tenfold cross-validated Pearson’s *r* =.62.

##### Language assessed for basic emotions

The basic emotion LBA models were pre-trained using a dataset in which a target word was presented with two random words and a synonym; first, the annotator had to select the correct synonym, and then annotate the target word for eight discrete emotions: anger, fear, anticipation, trust, surprise, sadness, joy, and disgust (Mohammad & Turney, 2013). Annotators used a four-point scale (“not”, “weakly”, “moderately”, “strongly”). “not” and “weakly” were interpreted as non-emotive regarding the target emotion, while “moderately” and “strongly” were categorized as emotive to the target emotion. A total of 38,726 assignments were made by 2216 annotators. In the analysis, we embedded the target words with their synonyms, predicting the different categories. The resulting LBA models demonstrated strong discriminative performance, with area under the ROC curve (*AUC*) values for classifying each target emotion as a multinomial model (*AUC* =.83).

#### Language visualizations

After developing the assessment models, we visualized the language significantly associated with the different conditions. We also created visualizations to understand differences in language responses between the online and offline samples. We used three techniques for making the visualizations.

##### Word comparison visualizations

First, word plots based on *Supervised Dimension Projection* were used for word comparison visualizations (Kjell et al., 2023). In short, supervised dimension projection plots compare responses from two groups (e.g., church versus remaining conditions), showing words significantly related to one group based on their position in the word embedding space. Since RoBERTa Large’s skewed distributions make it inappropriate for geometric plots (Reif et al., 2019), we used the Mixed Bread embeddings for these figures. The computations involve forming an *aggregated direction embedding* (i.e.*,* a comparative line on a linear scale through the embedding space). Each unique word is then projected onto this line by calculating the dot product between positioned embeddings of all individual words and the aggregated direction embeddings (i.e., supervised dimension projections). We used a permutation method to compute *p* values for each dot product for each static word embedding (here, we made 500 permutations). We applied false-discovery rate corrections for multiple comparisons (for more information, see Kjell et al., 2023).

To analyze language related to one condition, we grouped that condition as the first group and the remaining two conditions as the second group. After repeating this for all conditions, we visualized only the language positively associated with each condition. We also conducted additional linguistic inquiry and word count (LIWC) comparisons, available in the Supplemental Material (Table S12).

##### Topic modeling

The second visualization technique was topic modeling. Topics are clusters of words that often occur in similar contexts. We used the algorithm latent Dirichlet allocation (LDA; Blei et al., 2003) to create the topics, a probabilistic model that uncovers latent topics within text collections. We used both the online and offline data to create the topics. We allowed for both 1-grams and 2-grams (1-grams are single words and 2-grams are two words occurring together, e.g., “not_happy”). We excluded all words that appeared less than three times, created 25 topics following the rule of thumb to have 50 documents per topic (we had 1153 responses; Eichstaedt et al., 2021), and iterated the LDA algorithm 1000 times. For these first two techniques, we excluded stop words defined by the snowball lexicon (Benoit et al., 2018), as these words do not carry much meaningful information without any context, and LDA handles very common words poorly (Eichstaedt et al., 2021). We then compared the topics in each condition similarly to how we did in the supervised projection plot, where we grouped one condition as the first group and the remaining two conditions as the second group.

##### Response probability

The third and final technique involved showing the responses with the highest probability of being in each condition, as identified in our multinomial logistic regressions on the online holdout. In the multinomial logistic regressions, we assigned each response out-of-sample probabilities for each of the three conditions. We simply selected those responses with the highest probabilities of being in each condition. We transformed the probabilities into log odds, which range from – 4.6 to 4.6 when the probability range is 1 to 99% but can theoretically reach infinity. A log-odds of 0 means the response has a 50% probability of being from a certain condition. We also used this for the logistic regression models to estimate the probability that responses came from the online versus offline sample.

### Statistical tools

The analyses were computed in R (v4.2.2; R Core Team, 2022) using RStudio (v2023.3.0.386; Posit team, 2023). The following R-packages were used: *car* (Fox & Weisberg, 2019), *caret* (Kuhn et al., 2020), *corrplot* (Wei & Simko, 2021), *furrr* (Elsey, 2021) *ggcorrplot* (Kassambara, 2022), *ggpubr* (Kassambara, 2023a, b), *GPArotation* (Bernaards & Jennerich, 2005), *magrittr* (Bache et al., 2022), *psych* (Revelle, 2023), *purrr* (Mailund, 2022), *readxl* (Wickham & Bryan, 2019; Wickham et al., 2019), *rstatix* (Kassambara, 2023a, b), *text* (v1.4, Kjell et al., 2021a, b, c), *tidyverse* (Wickham & Bryan, 2019; Wickham et al., 2019), *topics* (Ackermann et al., 2024; Eijsbroek et al., 2025a, b).

## Results

### Randomization and manipulation checks

To examine the experiment's internal validity, we conducted a randomization and manipulation check. The randomization check assessed whether pre-intervention affect measures alone could classify the experimental conditions. As expected, the models performed poorly using both PANAS ratings and language-assessed valence (AUCs <.57), indicating negligible baseline differences between groups (see Supplemental Material Table S2). Further, the manipulation check confirmed that the experimental conditions affected participants’ affect, as shown by significant time × condition interaction effects for PANAS ratings (*F*_(2,739)_ = 29.84, *p* <.001, generalized *eta*^2^ =.01) and language-assessed valence (*F*_(2,739)_ = 30.97, *p* <.001, generalized *eta*^2^ =.02). A full correlation table between post-intervention measures can be found in Supplemental Material Table S3.

#### Data-driven classification models capture changes caused by the mood induction

**Fig. 2:**
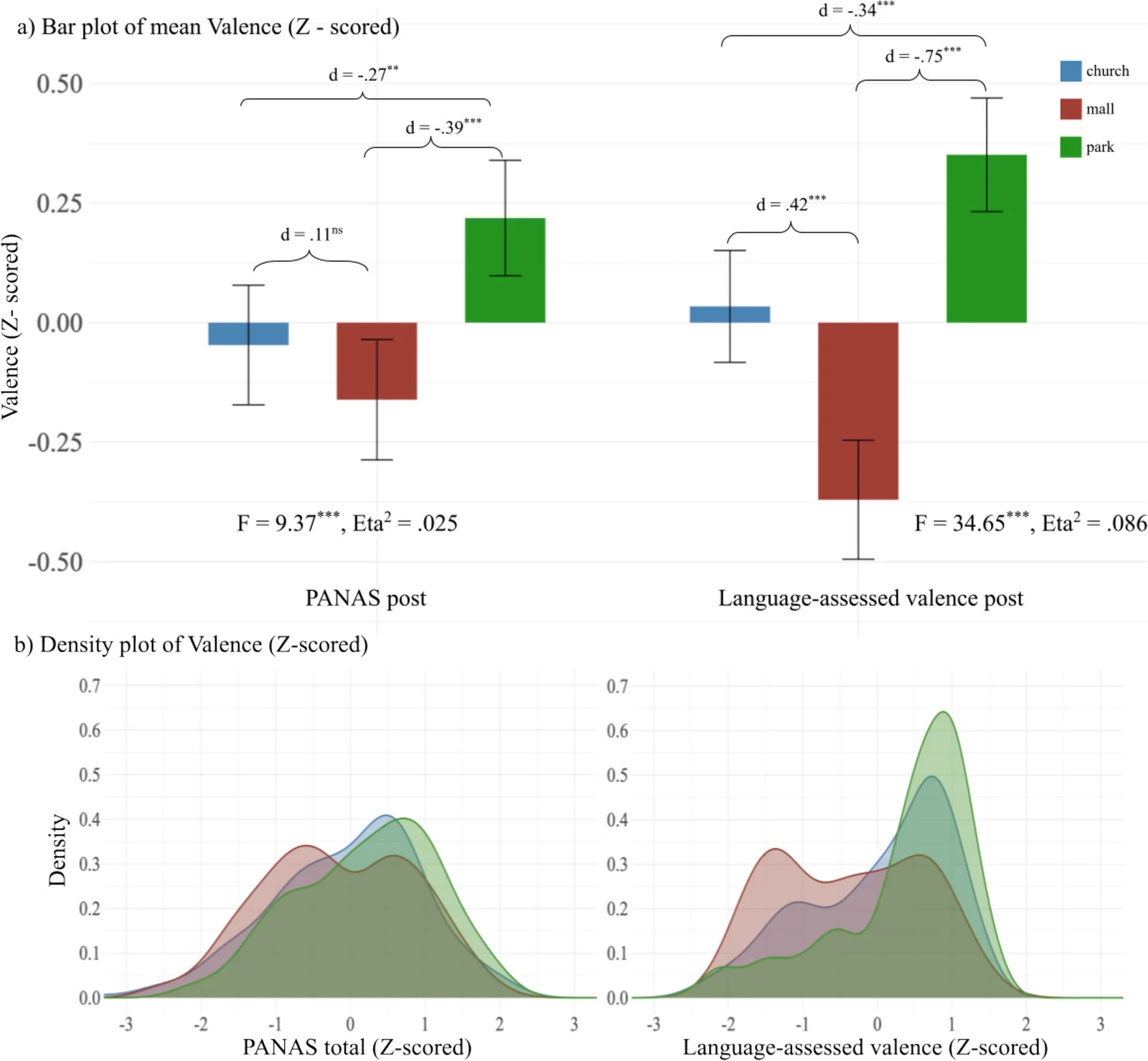
**a, b** Language-based assessments and PANAS comparison between the experimental conditions. *Note.* **a)** Bar plots of the *z*-scored PANAS and z-scored language-assessed valence based on the post-induction affect descriptions. *Error bars* are the 95% CI. *F* = Fisher’s one-way ANOVA test. *Eta*^*2*^ = generalized Eta-squared. *d* = Cohen’s *d*. ^ns^ = non-significant, * = *p* <.05, ** = *p* <.01, *** = *p* <.001. *N* = 739. **b)** Density plots showing the distributions of valence (*Z*-scored) of the three conditions. The color categories are the same across figures

The LBA of affect significantly outperformed PANAS in capturing changes in affect caused by the experimental manipulation (Table 2; Fig. 2). We systematically evaluated 28 PANAS-based models and 8 LBA models using multinomial ridge regression (see Supplemental Material Tables S4–S5). The best-performing LBA model used high-dimensional language embeddings from the *Mixed Bread Large* LLM, combining representations from participants’ pre-, post-, and difference responses. This model achieved an AUC of.74 [CI =.69 to.80] on the online holdout and.67 [CI =.60 to.73] on the offline holdout. In comparison, the best-performing PANAS model, which concatenated all 20 pre-, 20 post-induction items, and the difference score between the two items (60 dimensions), reached an AUC of.72 [CI =.65 to.78] on the online holdout but failed to generalize to the offline sample (AUC =.39, [CI =.33 to.46]). These findings indicate that LBA of affect shows higher causal validity than affect ratings. Furthermore, robustness checks, i.e., removing contextual words from the data, only marginally decreased the classification accuracy of the LBA (down to.65, [CI =.58 to.71]; see Tables S6–7 and Appendix A).

**Table 2:** Evaluating the best-performing models combining language and PANAS for classifying conditions

	Dimensions	AUC		
		CV	Holdout	
			Online	Offline
Best-performing models based on performance in the online holdout set:				
1. Best PANAS model^1^	60	.65^***^	.72^***^	.39^**^
2. Best embedding model^2^	3072	.73^***+++^	.74^***^	.67^***++^
3. Embeddings + PANAS	3112	.75^***+++^	.75^***^	.67^***+++^
Best-performing models based on performance in the offline holdout set:				
1. Best PANAS model^3^	1	.53^*^	.58^**^	.53^ns^
2. Best embedding model^4^	3072	.68^***+++^	.72^***+++^	.69^***+++^
3. Embedding + PANAS	3073	.67^***+++^	.72^***+++^	.69^***+++^

CV = cross-validated, AUC = area under the ROC curve. PANAS = Positive and Negative Affect Schedule. N_training_ = 586; N_online and offline holdout_ = 153

^1^ = Individual positive and negative affect ratings from pre- and post-induction, and difference score

^2^ = Pre-, post-, and difference embeddings from Mixed Bread

^3^ = Composite of negative affect ratings from post-induction

^4^ = Pre-, post-, and difference embeddings from RoBERTa

^*^ *= p* <.05, ^**^ *= p* <.01, ^***^ = *p* <.001 indicating whether AUC significantly differs from.50. ^ns^ = not significant; ^+^ indicates whether models in row 1 significantly differ from models' performance in other rows using bootstrapping sampling corrected for multiple comparisons using false discovery rate. ^+++^ *= p* <.001, ^++^ *= p* <.01

When models were evaluated on the offline holdout dataset, the best PANAS model (a single post-induction negative affect composite score) achieved a significant AUC of.53 (*p* >.05, [CI =.47 to 60]). In contrast, the equivalent LBA model achieved an AUC of.69 (CI =.62 to.75)—highlighting its robustness across settings. Further, combining PANAS ratings with affect description embeddings did not significantly improve model performance, suggesting that the LBA models captured almost all of the variance that ratings did.

### Theoretically grounded models capture changes in affect caused by the experimental conditions

Pre-trained LBAs of valence provided a theoretically grounded measurement that significantly outperformed PANAS ratings in distinguishing experimental conditions based on post-induction affect descriptions (Table 3; Fig. 2). The bar plots show that language-assessed valence produced substantially larger between-condition differences (*F* = 34.65, generalized eta^2^ =.086) compared to PANAS ratings (*F* = 9.37, generalized eta^2^ =.025). Effect size estimates (Cohen’s *d* for LBA comparisons =.34 [CI =.24 to.61],.75 [CI =.55 to.96], and.43 [CI =.16 to.53] versus Cohen’s *d* for PANAS comparisons.27 [CI =.10 to.45],.39 [CI =.22 to.57] and.11 [CI =.06 to.29]) further demonstrated stronger contrasts among conditions for language-assessed valence, particularly in differentiating the park and mall conditions (Cohen’s *d =.*75 versus.39). The density plots also illustrate clearer separation of distributions by condition in the LBAs of valence compared to PANAS (Fig. 2b).

**Table 3:** Evaluation of pre-trained LBAs and PANAS on classifying conditions based on post-induction affect descriptions

Assessment	Classifying conditions (church, mall, park) AUC	
	Online	Offline
	(*N* = 153)	(*N* = 153)
PANAS^1^	.59^*^	.52^ns^
*Language assessed for affect dimensions*		
Valence	.68^***+^	.63^***++^
Arousal	.61^*^	.58^ns^
*Language assessed for basic emotions*		
Anger	.66^***+^	.64^*++^
Anticipate	.50^ns^	.53^ns^
Disgust	.59^*^	.58^ns^
Fear	.55^ns^	.62^*++^
Joy	.61^*^	.62^**+^
Sadness	.56^ns^	.58^**^
Surprise	.55^ns^	.54^ns^
Trust	.65^***^	.63^**+^

The pre-trained large language model is Mixed Bread. Best valence and arousal model: Valence and arousal trained from Warriner et al. (2013; for other valence and arousal models see Table S8). More model assessments are in Supplemental Material Table S7. The basic emotions model is trained based on Mohammad & Turney (2013). No removal of any words. ^ns^ = not significant, ^*^ *= p* <.05, ^**^ *= p* <.01, ^***^ = *p* <.001 indicating whether AUC significantly differs from.50. ^+++^ *=p* <.001, ^++^ *=p* <.01, ^+^ *=p* <.05 compared to the PANAS model

^1^ = Post-intervention PANAS total score

Table 3 provides further detail on these theoretically informed assessments. In addition to valence, the discrete emotion anger showed significantly higher AUC compared to PANAS for both the online and offline holdout sets (*p* <.05, AUC.66 online to.64 offline). LBAs of arousal and the discrete emotions of disgust, joy, and trust also showed significant discrimination of experimental conditions in both the training and online holdout sets (*p* <.05; AUCs =.59 to.65). While their AUCs were consistently on par with or above those of PANAS (which had AUC =.59), these differences were not statistically significant (*p* >.05). In the offline sample, the emotions fear, joy, sadness and trust showed significant AUCs (*p* <.05; AUCs =.58 to.63) while the PANAS composite score did not reach significance. More visualizations of the comparisons together with confusion matrices are available in the Supplemental Material (Figs. S1–S3, Table S13).

Overall, these results demonstrate that theoretically grounded LBAs possess at least as strong causal validity compared to traditional rating scales. These findings highlight the potential value of pretrained, theory-informed LBA models for capturing subtle, contextually induced changes in affective states that may be less accessible to closed-ended rating scales.

### Language responses reveal condition-specific affective profiles

**Fig. 3:**
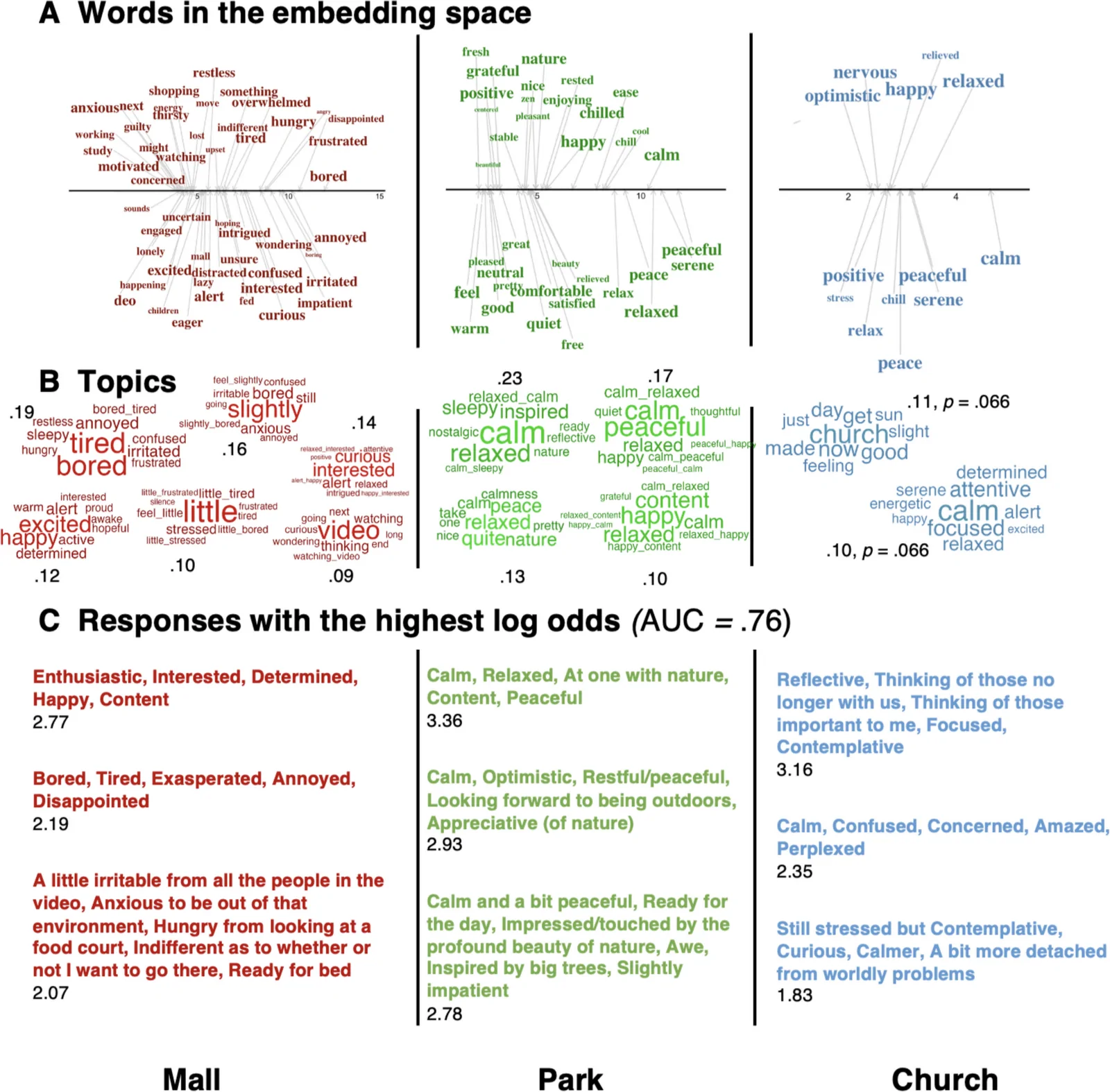
Language significantly associated with each condition versus the remaining conditions. *Note.* *AUC* = area under the ROC curve. Panels A and B used the entire online data (*N* = 739). Panel C uses the predictions on the online holdout (*N* = 153). For panel A, the *x*-axis represents the dot product, where higher scores mean stronger associations with the condition. The *y*-axis has no meaning and is only used for visualization. For panel B, the topics are sorted from the strongest associated topic (***upper left***) to the weakest associated topic (***bottom right***). All responses in panel C were correctly classified. Condition-specific (i.e., church, mall, or park) conditions were not excluded from visualization analyses

Participants’ post-induction affect descriptions showed distinct, condition-specific affective patterns, demonstrating that open-ended responses can capture nuanced emotional shifts aligned with the experimental manipulation. Figure 3 visualizes these significant, differentiated words and topics using three complementary techniques, which we interpreted through the Circumplex Model of Affect (Russell, 1980a, b). In panel A, words significantly associated with each condition in the online data (compared to the other conditions) are displayed. The mall condition was characterized by both low- and high-arousal negative affect (e.g., *bored*, *indifferent*, *annoyed*, *irritated*), physical needs (e.g., *hungry*, *thirsty*), and high-arousal positive affect (e.g., *excited*, *alert*). In contrast, the park and church conditions predominantly featured low-arousal positive affect words (e.g., *serene*, *calm*, *peaceful*). The park condition included more low-arousal positive descriptors overall, while the church condition also included some negative affect terms (e.g., *stress*, *nervous*).

Panel B shows the topics significantly associated with each condition. The topics showed a similar pattern to the significant words, with negative affect (e.g., *tired*, *slightly bored*, and *little stressed* topics) in the mall conditions, as well as high-arousal positive affect (e.g., *excited*, *interested*, and *alert* topics). Again, all topics significantly related to the park condition involved low-arousal positive affect (e.g., *calm*, *relaxed*, and *peaceful* topics). For the church condition, one topic involved positive affect (*calm*, *focused*, *alert*), and one was a church topic. Overall, for both panels A and B, more language was associated with the mall condition, followed by the park, and lastly, the church.

Lastly, panel C shows the responses that the best-performing multinomial logistic regressions identified as having the highest probabilities for being in each condition. They generally supported the previous results, with mall responses including low- and high-arousal negative affect (e.g., Bored, Tired, Exasperated, Annoyed, and Disappointed) and high-arousal positive affect (e.g., *Enthusiastic, Interested, Determined, Happy,* and *Content*). The park responses involved mostly low-arousal positive affect, with all three responses starting with “*Calm”*. The church responses showed a tendency toward broad reflection (e.g., “*Thinking of those no longer with us”* and “*A bit more detached from worldly problems”*). Overall, the mall language involved low- and high-arousal negative emotions and high-arousal positive emotions, and these signals were stronger than the signals significantly related to the park and church conditions (i.e., the mall language generated more significant words and topics). Both the park and church language involved low-arousal positive emotions, with a bit more of this signal in the park condition. The church condition showed tendencies to invoke deeper reflections about life.

### The difference between online and offline language

**Fig. 4:**
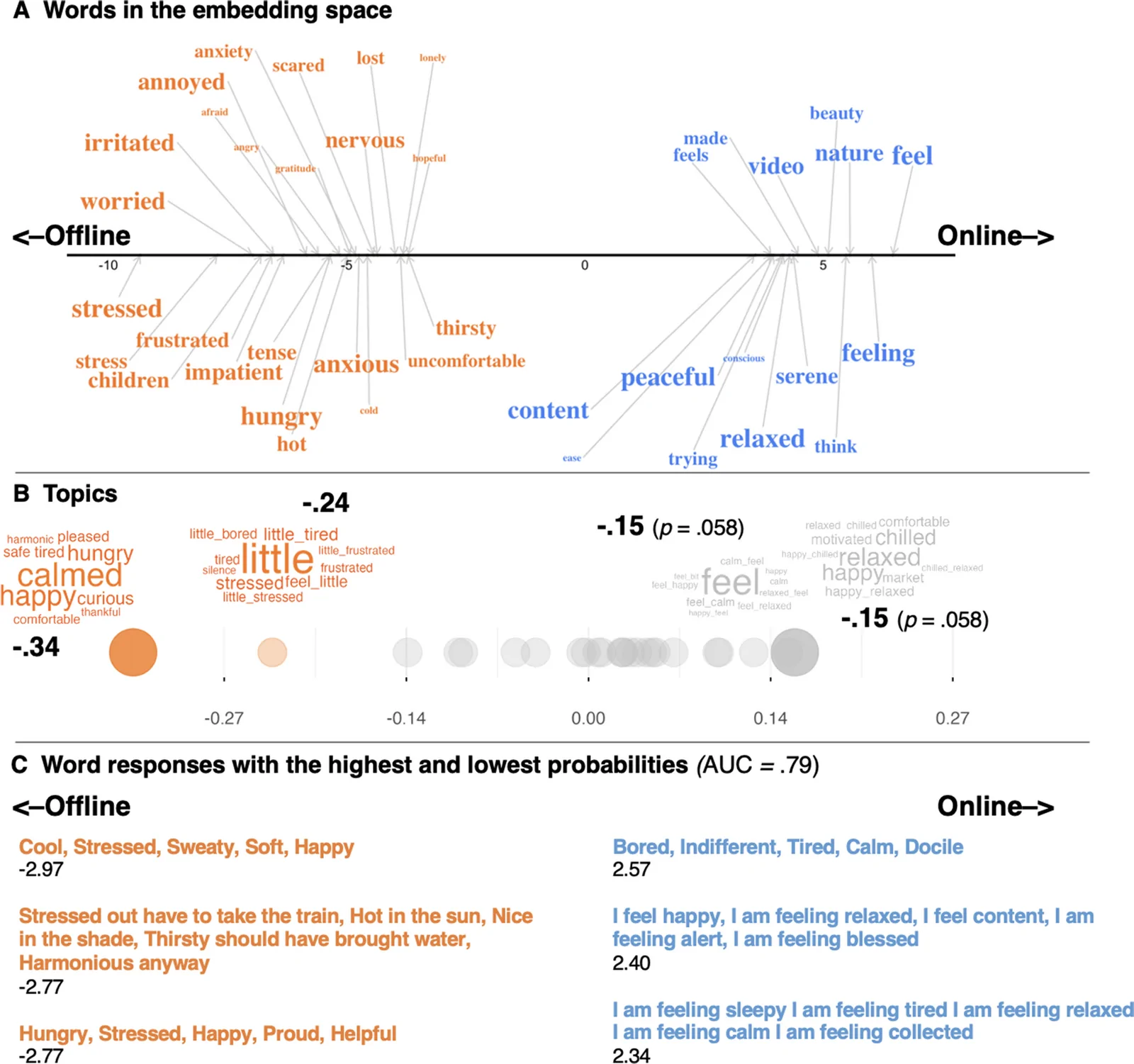
Language significantly associated with the offline versus online samples. *Note.* *AUC* = area under the ROC curve. The data are only from the offline and online holdouts, meaning each study setting has 153 participants. For panel A, the *x*-axis represents the dot product, where higher scores mean stronger associations with the condition. The *y*-axis has no meaning and is only used for visualization. For panel B, the topics are sorted from the strongest associated offline topic (to the left) to the strongest associated online topic (to the right). Gray topics were non-significant after correction for multiple corrections. All responses in Panel C were correctly classified. Condition-specific (i.e., church, mall, or park) conditions were not excluded from visualization analyses

Given the relatively poor generalization from the online-trained models to the offline holdout—observed for both LBA and rating-based models—we investigated how the language differed between the two samples. As shown in Fig. 4, the language patterns between online and offline participants were clearly distinguishable, with a classification model accurately separating them (AUC =.79, *p* <.001). Offline data featured words and topics related to immediate, visceral, or physical states such as “*stressed”*, “*irritated”*, and “*hungry”*, which often reflect real-world pressures or challenges. In contrast, online data predominantly featured words and topics associated with reflective, affective states, including “*peaceful”*, “*feeling”*, and “*beauty”*. This divergence suggests that visiting the offline locations generated language tied to situational stressors and physical experiences, while the online contexts induced more abstract, idealized, or affective states. The distinction was further supported by the specific responses identified as having the highest probability of being in each setting, where one of the offline responses described specific stress-inducing scenarios (“*Stressed out have to take the train”*), whereas online responses focused on inner calm and readiness, aligning with the tendency of digital platforms to foster reflective and curated expressions (“*I feel content”*). These patterns underscore the importance of context in interpreting linguistic data from different sources and its implications for understanding affective and cognitive states. They also highlight the limitations of the generalizability of mood induction procedures across online and offline settings.

## Discussion

This study evaluated the causal validity of language-based assessments of affect compared with a traditional rating scale, using a randomized experimental *mood induction procedure*. In line with our pre-registered hypotheses, data-driven classifications using LBAs more accurately identified participants’ experimental conditions than the rating scale. Beyond superior predictive performance, theoretically grounded pre-trained LBAs of affect dimensions and emotions, along with data-driven language visualizations, provided interpretable insights into how descriptions of individuals’ affective experiences varied across conditions and platforms (online vs. offline).

While prior research has demonstrated strong convergence between LBAs and rating scales, as well as robust criterion validity in predicting external behaviors and psychological outcomes (Eichstaedt et al., 2018; Gu et al., 2024; Kjell et al., 2019; Nilsson et al., 2024a; Schwartz et al., 2014; Son et al., 2023), this study is—to our knowledge—the first to provide supporting evidence for the causal validity of LBAs.

### Data-driven language-based classifications

The higher classification accuracy of LBAs supports their ability to sensitively capture condition-induced change in affect beyond the corresponding rating. Our systematic evaluation showed that even when combined with PANAS, the best LBA models experienced either negligible improvement or only minor gains in AUC (e.g., an increase of.01), indicating that the linguistic embeddings already encapsulated nearly all the predictive signal from the rating data. However, instead of producing a single interpretable scale score (e.g., level of positive affect), these data-driven classification models generate probabilistic scores reflecting the likelihood that a person participated in a specific experimental condition (e.g., park vs. mall). While inherently context-dependent and not directly comparable to continuous affect scores, the classification metric (AUC) provides an interpretable effect size for how well language-based affect descriptions distinguish between conditions. To move beyond raw accuracy and illuminate the specific emotional content underlying these differences, we applied theoretically grounded, pre-trained LBA models of affect dimensions and basic emotions as well as data-driven language-based visualizations.

### Theoretically grounded language-based assessments

Whereas the data-driven classification models operate without predefined emotional frameworks, the theoretically grounded, pre-trained LBAs were explicitly informed by psychological models of affect and emotion. By quantifying emotional content along established dimensions—such as valence and arousal from dimensional theories (Russell, 1980a, b), and discrete categories like joy, anger, and trust from basic emotion theories (Ekman, 1992)—these models directly reflect theoretical constructs from the science of affect and emotion. For example, participants in the park condition expressed significantly higher positive valence than those in the mall condition—aligning with prior research showing that natural environments reliably evoke more positive emotions than urban settings (e.g., Bratman et al., 2012; Kuo, 2015). Notably, language-assessed valence and the discrete emotion anger revealed larger differences between experimental conditions than PANAS ratings.

### Data-driven language-based visualizations

The data-driven language-based visualizations also provided insights that go beyond raw classification accuracy. Unlike the theoretically grounded pretrained models, these visualizations are not constrained by predefined emotion categories but instead reveal significant, condition-specific patterns emerging from participants’ affect descriptions. The results are largely consistent with the patterns observed in the theoretically grounded valence analyses: For example, language in the park and church conditions reflected higher levels of low-arousal positive emotions, lower levels of high-arousal positive emotions, and less negative emotion, suggesting a calming influence of these settings as evident from the significant words and topics identified. Moreover, our observation that significantly “more language” (a higher volume of unique words and topics) was associated with the mall, we argue, highlights the complex, mixed affective states that can be triggered by a modern consumer environment. The mall environment simultaneously evoked high-arousal negative affect (irritation), low-arousal negative affect (boredom), and visceral physiological needs (hunger/thirst). Open-ended language captures this multidirectional emotional friction, showing that real-world affect is rarely univalent or isolated from immediate surroundings.

The data-driven visualizations helped explain differences in generalizability between the online and offline samples. The offline participants used more contextually grounded language, linking emotions to specific real-world states such as “*Stressed to take the train*”, while the online sample was overall more reflective in its emotional language, such as “I am feeling blessed”.

Similarly, the finding that the church condition invoked distinct “deeper reflections about life” (e.g., thoughts of missing loved ones or feeling detached from worldly problems) offers more conceptual clarity. This demonstrates that language does not merely report an internal state; it actively shapes and categorizes it. In this case, the church did not just induce a passive, low-arousal positive state (such as simple relaxation), but actively directed participants toward an existential, cognitive frame of reference. These insights underscore the utility of data-driven language analyses not only for measuring affective change but also for explaining and contextualizing those changes in interpretable ways.

### Causal validity: Affect, context, and dimensionality

Taken together, these findings support the causal validity of LBAs of affect, demonstrating their capacity to detect affective changes resulting from a randomized experimental manipulation. While one could argue that the superior classification performance of LBAs might be driven by contextual cues (e.g., “*Hungry from looking at a food court*”) rather than genuine affective states (e.g., “*Enthusiastic, Interested, Determined, Happy, Content*”), several lines of evidence counter this concern. First, stricter filtering procedures that removed contextual references led to only marginal reductions in classification accuracy (see Supplemental Material Table 7), suggesting that affective information—not just situational content—drives model performance. Second, the theoretically grounded LBAs rely not on context-specific words, but on affective dimensions (valence and arousal) and probabilities of discrete emotions derived from established language models. Third, data-driven language visualizations revealed psychologically meaningful differences in emotional tone across conditions—such as calmness in park settings and agitation in malls—that extended beyond surface-level context.

Importantly, we argue that emotional experiences are rarely detached from context. People naturally embed affect in real-world narratives (e.g., “*At one with nature*”), linking feelings to environments, events, and social settings (Barrett, 2017; Kals et al., 1999; Kjell, 2011). From this perspective, contextual language is not semantic noise but a source of ecological validity, offering insight into how affect is genuinely experienced and expressed. A notable finding was the reduced performance between the online and offline holdout, particularly for the PANAS model. The discrepancy likely reflects the effect of surrounding real-world context, highlighted by the different language patterns: more direct, physical emotional language in the offline sample versus more inner, reflective emotional language in the online sample. On a closed-ended rating scale, these real-world factors may introduce noise (e.g., travel stress), compressing response variance and obscuring the subtle signal of the experimental induction. LBAs, by contrast, remained more robust, arguably because the open-ended format allowed participants to express this emotional context. However, although the results give some preliminary hints, we do not know to what degree emotions are embedded in the local context, which future research could examine.

Participants describe their emotional states freely, which may allow the method to capture nuances missed by fixed-item scales. The open-ended responses are then transformed into high-dimensional embeddings that capture affect across thousands of word embedding dimensions. This expressive and computational flexibility may allow LBA to more accurately reflect the complexity and personal relevance of real-world emotional experiences (Boyd & Schwartz, 2021; Kjell et al., 2024). Notably, increasing dimensionality improved rating accuracy (using PANAS as 20 single items), indicating that higher dimensionality generally increased accuracy. However, the 1068 dimensions of LBAs used in this study can rarely be translated into fixed-item scales, which often use only a few items for a single psychological construct.

## Limitations and future direction

The present study has some limitations that should be considered when interpreting the findings. First, the data are linked to two separate pre-registrations. The first pertains to a Master’s thesis and the offline sample, which was not followed in this paper (the pre-registration and thesis are available at https://osf.io/97cuj). The second pre-registration was specific to this study and guided the online data collection. We made two deviations: First, because computer science has advanced rapidly, we adjusted the models included to capture the latest developments. The pre-registered models are still part of the Supplemental Material in Table S5, further providing support for our hypotheses*.* Second, one condition (a video of a market) was excluded from the online sample to harmonize the offline and online data. The Supplemental Material (Table S9) includes additional analyses using all four conditions, further supporting the superiority of the accuracy of LBAs over rating scales. Further pairwise comparisons for LBAs and PANAS can also be found in Supplemental Material (Tables S10–11).

Second, the current study aims for high ecological validity by examining mood inductions through experiences of common urban environments, where the environments for the conditions were chosen partly based on the physical closeness to each other. Rather than explicitly targeting positive or negative emotions, as the PANAS is designed to capture, the present experiment focuses on more naturalistic, context-driven mood shifts. To more directly assess the sensitivity of PANAS and comparable rating scales, future studies should aim to induce specific emotional states—such as positive affect—using different targeted environments, and evaluate how well models perform relative to traditional scales under those conditions.

Third, while the pre-trained language-based models of valence, arousal, and basic emotions in this study offer theoretically grounded assessments of affect, their construct validity warrants continued evaluation. Although our findings show alignment with experimental manipulations and prior theory, further validation against behavioral outcomes, physiological measures, and longitudinal data would strengthen confidence in their broader applicability. We encourage future studies to benchmark these models against multimodal indicators of affect to clarify their psychometric properties. Importantly, all models and code used in this study are openly available through the *text* package (Kjell et al., 2023) and the language-based assessment model (L-BAM) library (Nilsson et al., 2026), enabling transparent application and replication by other researchers.

Fourth, the reliance on English-language embeddings and the translation of the Swedish offline sample represent a potential ‘cultural bottleneck’. Emotional language is deeply embedded in cultural and contextual frameworks (Hoemann, 2023); therefore, the affective language captured by our models may not be universally representative of all linguistic groups (Blasi et al., 2022). While independent researchers verified our translation process, the English-centric nature of many state-of-the-art LLMs remains a ‘bottleneck’ for the field. Future research should use multilingual LLMs and culturally broader samples to test whether these affective linguistic signatures are invariant across cultural contexts.

We acknowledge that the current results suggest LBAs are currently better suited for research and group-level analysis rather than individual clinical diagnostics. However, this performance should be viewed as a promising baseline for future models that may incorporate even larger training datasets and more nuanced and diverse contexts. One promising area is ecological momentary assessment, where LBAs can capture subtle, in-the-moment affective shifts from open-ended responses in naturalistic settings, helping bridge the gap between traditional laboratory measures and everyday emotional regulation within its natural habitat (Kaplan et al., 2022). Furthermore, recent technological innovations provide secure mobile frameworks to collect spoken or written responses longitudinally, yielding richer verbal data and stronger psychometric signals than traditional text-entry surveys (Kaplan et al., 2025). Another is intervention research—particularly in the domain of well-being and happiness. Although interventions such as exercise, meditation, or time in nature are widely promoted, replication failures are common (Folk & Dunn, 2023, 2024), raising questions about their effectiveness. One possible reason is that traditional rating scales may overlook the diverse ways people experience and express positive affect. Because LBAs can detect nuanced and contextually grounded emotional expressions, they could enhance the evaluation of happiness interventions by revealing how and why affective states change—or fail to change—across individuals and contexts. Future studies could combine LBAs with rating scales to offer a more comprehensive and ecologically valid picture of affective change.

## Conclusion

This study provides evidence for the causal validity of language-based assessments in detecting changes in affect and emotion resulting from experimental manipulation—outperforming the traditional affect rating scale PANAS. By leveraging open-ended language, LBAs can capture condition-specific differences in affective expression within the present experimental context, offering richer descriptive information than the rating scale used here. Unlike fixed-item questionnaires, they capture both subtle emotional shifts and the language individuals use to make sense of their experiences. The cross-platform comparison of LBA enables understanding of the biases and reasons for changes in performance across data collection contexts, otherwise masked with rating comparisons. These findings strengthen the scientific foundation of LBAs as valid tools for assessing affective change and highlight their potential for broader application in experimental research where understanding the richness of emotional experience is essential.

## Supplementary Information

Below is the link to the electronic supplementary material.

Supplementary file1 (DOCX 22 KB)

Supplementary file2 (DOCX 15 KB)

Supplementary file3 (DOCX 8 KB)

## Data Availability

This study was pre-registered on Open Science Framework (OSF; offline: https://osf.io/7uz29; online: https://osf.io/zj9p8), and study materials are also available on OSF.

## Footnotes

1 The authors did not have access to the data before the pre-registration was completed.

2 The experiment also included measures not used in the analyses of this paper, including: the Ten Item Personality Measure (TIPI; Gosling et al., 2003), open-ended questions regarding personality, daily activities, and voice recordings of a standardized question and of affect descriptions.

3 http://lup.lub.lu.se/student-papers/record/9133032
