# Supplementary material for: Language-based affect assessments capture experiment-induced changes beyond rating scales
Source: Behav Res Methods. 2026 Nov 3;58(12):340. doi: 10.3758/s13428-026-03200-z (PMC13736416; doi:10.3758/s13428-026-03200-z)
Supplement: Supplementary file 1 [file 13428_2026_3200_MOESM1_ESM.docx]

# Table s12 *Supplementary Linguistic Inquiry and Word Count (LIWC) analyses*

Cells report participant-level means with standard deviations in parentheses. Most LIWC category scores represent the percentage of words matching the dictionary. WC is word count, WPS is words per sentence, and Analytic, Clout, Authentic, and Tone are LIWC summary scores. Variables containing only missing or zero values within a sample were omitted.

# 1. Online sample

Church N = 254; Mall N = 248; Park N = 237

## 1.1. Text summary and linguistic coverage

| **LIWC category** | **Church** | **Mall** | **Park** |
| --- | --- | --- | --- |
| Word count | 12.74 (13.08) | 12.37 (11.97) | 12.61 (12.09) |
| Analytic | 66.87 (36.93) | 66.53 (34.37) | 66.37 (34.32) |
| Clout | 8.66 (17.32) | 4.54 (6.21) | 6.24 (15.13) |
| Authentic | 62.56 (36.70) | 62.49 (34.78) | 69.56 (34.33) |
| Tone | 65.99 (44.26) | 45.42 (46.72) | 78.87 (37.96) |
| Words per sentence | 11.55 (11.11) | 10.68 (8.96) | 10.92 (9.61) |
| Big words | 40.85 (23.45) | 41.22 (22.19) | 39.47 (22.08) |
| Dictionary words | 92.15 (10.10) | 91.03 (10.79) | 90.38 (11.16) |
| Linguistic | 65.71 (19.21) | 62.12 (20.98) | 63.81 (18.25) |

## 1.2. Function words and grammar

| **LIWC category** | **Church** | **Mall** | **Park** |
| --- | --- | --- | --- |
| Function words | 20.82 (22.55) | 20.93 (22.49) | 20.57 (21.62) |
| Pronoun | 5.48 (9.78) | 4.94 (8.34) | 5.44 (9.42) |
| Personal pronouns | 4.73 (9.07) | 3.52 (7.39) | 4.79 (8.95) |
| I | 4.64 (9.01) | 3.52 (7.38) | 4.76 (8.95) |
| We | 0.04 (0.42) | 0.00 (0.00) | 0.02 (0.28) |
| You | 0.01 (0.08) | 0.00 (0.00) | 0.02 (0.25) |
| She/he | 0.04 (0.42) | 0.00 (0.00) | 0.00 (0.00) |
| They | 0.01 (0.11) | 0.00 (0.07) | 0.00 (0.00) |
| Impersonal pronouns | 0.75 (2.13) | 1.41 (3.29) | 0.64 (2.22) |
| Determiners | 4.75 (7.33) | 5.43 (7.78) | 4.71 (7.28) |
| Article | 3.11 (5.62) | 3.50 (5.80) | 2.74 (4.86) |
| Number | 0.03 (0.32) | 0.04 (0.38) | 0.13 (1.17) |
| Prepositions | 6.09 (8.67) | 5.48 (8.08) | 6.09 (8.17) |
| Auxiliary verbs | 2.51 (5.80) | 2.90 (5.97) | 2.09 (4.86) |
| Adverb | 2.02 (4.71) | 2.35 (5.02) | 1.86 (4.36) |
| Conjunctions | 1.49 (3.58) | 1.59 (4.15) | 1.77 (4.02) |
| Negations | 0.47 (1.95) | 0.66 (2.94) | 0.47 (2.24) |
| Verb | 7.69 (10.99) | 7.45 (10.51) | 8.73 (11.22) |
| Adjectives | 39.12 (24.92) | 36.01 (23.21) | 36.21 (22.59) |
| Quantity | 3.17 (6.22) | 3.58 (6.88) | 2.78 (6.03) |

## 1.3. Affect and cognition

| **LIWC category** | **Church** | **Mall** | **Park** |
| --- | --- | --- | --- |
| Cognition | 13.65 (13.39) | 12.36 (12.55) | 12.03 (12.38) |
| All-or-none terms | 0.19 (1.04) | 0.22 (1.68) | 0.31 (1.66) |
| Cognitive processes | 13.47 (13.35) | 12.13 (12.41) | 11.72 (12.31) |
| Insight | 7.29 (10.72) | 6.23 (9.44) | 7.18 (10.56) |
| Cause | 1.02 (3.71) | 0.73 (3.28) | 0.88 (3.37) |
| Discrepancy | 1.69 (4.84) | 1.13 (3.64) | 1.19 (3.44) |
| Tentative language | 2.64 (6.00) | 2.92 (7.16) | 1.20 (3.64) |
| Certitude | 0.18 (1.50) | 0.21 (1.43) | 0.05 (0.49) |
| Differentiation | 0.72 (2.88) | 1.11 (3.46) | 0.70 (2.54) |
| Memory | 0.25 (1.81) | 0.24 (1.85) | 0.32 (2.09) |
| Affect | 46.02 (27.15) | 44.40 (26.51) | 46.55 (26.86) |
| Positive tone | 31.33 (26.69) | 22.48 (24.02) | 37.00 (27.70) |
| Negative tone | 14.09 (20.12) | 21.34 (24.27) | 9.05 (16.91) |
| Emotion | 37.68 (24.92) | 37.44 (25.23) | 36.95 (24.09) |
| Positive emotion | 24.50 (22.81) | 17.58 (21.24) | 28.45 (23.66) |
| Negative emotion | 12.49 (18.31) | 19.29 (23.16) | 8.02 (15.63) |
| Anxiety | 5.08 (12.26) | 5.51 (10.77) | 2.66 (7.59) |
| Anger | 1.13 (4.76) | 2.86 (7.84) | 1.14 (5.27) |
| Sadness | 0.66 (3.04) | 1.00 (4.85) | 0.50 (2.90) |
| Swear | 0.00 (0.00) | 0.00 (0.00) | 0.03 (0.45) |

## 1.4. Social processes and motivational drives

| **LIWC category** | **Church** | **Mall** | **Park** |
| --- | --- | --- | --- |
| Drives | 3.49 (7.50) | 2.14 (5.69) | 2.12 (6.20) |
| Affiliation | 0.40 (1.84) | 0.32 (2.28) | 0.23 (1.88) |
| Achievement | 2.21 (5.86) | 1.53 (5.02) | 1.48 (4.76) |
| Power | 0.87 (3.89) | 0.27 (1.95) | 0.33 (2.96) |
| Social | 3.64 (7.37) | 3.23 (7.54) | 2.39 (5.60) |
| Social behaviour | 2.90 (6.81) | 2.80 (7.37) | 1.76 (4.97) |
| Prosocial behaviour | 0.67 (3.45) | 0.31 (2.91) | 0.64 (3.19) |
| Politeness | 0.14 (1.63) | 0.00 (0.00) | 0.00 (0.00) |
| Conflict | 0.39 (2.31) | 1.05 (4.82) | 0.24 (1.94) |
| Morality | 0.19 (1.72) | 0.39 (2.95) | 0.35 (2.58) |
| Communication | 0.03 (0.28) | 0.13 (0.78) | 0.09 (0.67) |
| Social referents | 0.42 (1.77) | 0.15 (1.04) | 0.16 (1.21) |
| Family | 0.11 (0.78) | 0.07 (0.89) | 0.01 (0.20) |
| Friend | 0.02 (0.18) | 0.00 (0.00) | 0.01 (0.22) |
| Female | 0.03 (0.26) | 0.01 (0.18) | 0.00 (0.00) |
| Male | 0.06 (0.52) | 0.01 (0.17) | 0.01 (0.20) |

## 1.5. Physical states, needs, perception, and time

| **LIWC category** | **Church** | **Mall** | **Park** |
| --- | --- | --- | --- |
| Physical | 5.24 (8.94) | 7.99 (11.84) | 5.72 (9.83) |
| Health | 3.44 (7.11) | 4.25 (7.36) | 3.25 (6.89) |
| Illness | 0.08 (0.86) | 0.19 (1.26) | 0.20 (1.43) |
| Wellness | 0.25 (2.08) | 0.04 (0.48) | 0.32 (2.14) |
| Mental | 0.08 (1.25) | 0.00 (0.00) | 0.00 (0.00) |
| Sexual | 0.00 (0.00) | 0.08 (1.27) | 0.00 (0.00) |
| Food | 0.60 (2.86) | 1.98 (6.02) | 0.51 (2.98) |
| Death | 0.08 (1.25) | 0.02 (0.32) | 0.00 (0.00) |
| Need | 0.77 (3.18) | 2.06 (5.94) | 0.56 (3.00) |
| Want | 1.23 (4.35) | 0.85 (3.35) | 0.86 (3.15) |
| Acquire | 0.37 (1.83) | 0.29 (1.64) | 0.47 (2.00) |
| Lack | 0.67 (3.06) | 1.99 (6.01) | 0.51 (2.76) |
| Fulfill | 0.30 (2.15) | 0.29 (2.08) | 0.32 (2.01) |
| Fatigue | 5.61 (10.78) | 9.12 (13.07) | 4.96 (9.65) |
| Reward | 0.48 (2.72) | 0.59 (3.09) | 0.71 (3.28) |
| Risk | 1.44 (5.54) | 2.95 (6.70) | 1.43 (4.62) |
| Curiosity | 3.82 (9.71) | 4.58 (10.27) | 3.19 (7.21) |
| Allure | 5.92 (9.90) | 4.64 (7.92) | 6.98 (9.79) |
| Perception | 13.00 (13.34) | 13.46 (14.57) | 12.86 (13.02) |
| Attention | 5.32 (10.57) | 7.02 (13.44) | 3.53 (8.69) |
| Motion | 0.42 (2.04) | 0.85 (2.61) | 1.20 (3.34) |
| Space | 2.21 (5.00) | 2.06 (4.61) | 3.04 (5.86) |
| Visual | 0.90 (3.54) | 0.52 (1.99) | 0.63 (2.46) |
| Auditory | 0.67 (3.01) | 0.79 (3.39) | 0.65 (3.01) |
| Feeling | 3.59 (7.76) | 2.61 (6.32) | 4.34 (8.43) |
| Time | 1.55 (3.81) | 1.58 (4.34) | 1.58 (4.10) |
| Past focus | 1.56 (4.49) | 1.67 (4.57) | 1.75 (5.14) |
| Present focus | 3.77 (8.42) | 2.82 (6.70) | 4.22 (8.54) |
| Future focus | 1.74 (5.71) | 1.48 (4.62) | 0.92 (3.57) |

## 1.6. Culture, lifestyle, conversation, and punctuation

| **LIWC category** | **Church** | **Mall** | **Park** |
| --- | --- | --- | --- |
| Culture | 0.00 (0.00) | 0.01 (0.17) | 0.12 (1.14) |
| Technology | 0.00 (0.00) | 0.01 (0.17) | 0.12 (1.14) |
| Lifestyle | 6.95 (8.93) | 4.86 (7.89) | 8.33 (9.17) |
| Leisure | 5.46 (8.05) | 4.05 (7.68) | 7.62 (8.76) |
| Home | 0.06 (0.45) | 0.08 (0.57) | 0.18 (1.11) |
| Work | 0.70 (3.02) | 0.43 (2.02) | 0.32 (1.98) |
| Money | 0.03 (0.32) | 0.23 (1.42) | 0.02 (0.27) |
| Religion | 0.72 (3.08) | 0.07 (0.66) | 0.22 (1.83) |
| Conversation | 0.14 (1.17) | 0.24 (1.91) | 0.29 (2.17) |
| Netspeak | 0.01 (0.13) | 0.02 (0.37) | 0.09 (1.13) |
| Assent | 0.14 (1.16) | 0.24 (1.91) | 0.20 (1.86) |
| All punctuation | 1.93 (6.05) | 2.48 (7.31) | 1.87 (5.65) |
| Period | 0.94 (4.99) | 1.09 (5.06) | 0.98 (4.42) |
| Comma | 0.39 (1.91) | 0.43 (2.86) | 0.15 (0.94) |
| Question marks | 0.00 (0.00) | 0.03 (0.53) | 0.01 (0.15) |
| Exclamation marks | 0.00 (0.00) | 0.18 (1.62) | 0.02 (0.20) |
| Apostrophes | 0.26 (1.30) | 0.27 (1.46) | 0.21 (1.30) |
| Other punctuation | 0.34 (2.35) | 0.48 (2.72) | 0.50 (2.82) |

# 2. Offline sample

Church N = 45; Mall N = 54; Park N = 54

## 2.1. Text summary and linguistic coverage

| **LIWC category** | **Church** | **Mall** | **Park** |
| --- | --- | --- | --- |
| Word count | 8.67 (6.22) | 12.22 (10.91) | 11.15 (11.27) |
| Analytic | 75.23 (36.50) | 80.69 (30.79) | 76.46 (34.29) |
| Clout | 15.59 (25.11) | 18.17 (20.30) | 10.80 (19.35) |
| Authentic | 60.81 (34.60) | 62.55 (36.16) | 64.23 (30.65) |
| Tone | 65.12 (45.61) | 43.13 (45.27) | 81.29 (35.40) |
| Words per sentence | 7.63 (4.13) | 10.71 (8.30) | 10.13 (9.08) |
| Big words | 35.18 (19.64) | 43.73 (24.30) | 35.62 (20.69) |
| Dictionary words | 93.98 (10.37) | 91.74 (10.80) | 90.68 (15.89) |
| Linguistic | 66.00 (20.36) | 60.98 (23.57) | 66.23 (21.24) |

## 2.2. Function words and grammar

| **LIWC category** | **Church** | **Mall** | **Park** |
| --- | --- | --- | --- |
| Function words | 16.81 (19.33) | 20.24 (23.56) | 20.41 (21.98) |
| Pronoun | 1.32 (3.70) | 3.13 (6.63) | 2.59 (6.69) |
| Personal pronouns | 0.82 (2.57) | 1.98 (4.18) | 1.87 (6.18) |
| I | 0.68 (2.44) | 1.98 (4.18) | 1.87 (6.18) |
| They | 0.14 (0.93) | 0.00 (0.00) | 0.00 (0.00) |
| Impersonal pronouns | 0.51 (2.16) | 1.16 (2.95) | 0.72 (2.17) |
| Determiners | 7.06 (10.16) | 6.22 (8.58) | 7.46 (9.74) |
| Article | 4.48 (7.29) | 4.10 (6.29) | 4.42 (7.48) |
| Number | 0.00 (0.00) | 0.00 (0.00) | 0.15 (1.13) |
| Prepositions | 4.37 (7.37) | 7.92 (9.43) | 4.87 (7.46) |
| Auxiliary verbs | 1.14 (3.29) | 1.58 (3.98) | 2.02 (5.88) |
| Adverb | 2.24 (6.43) | 2.13 (4.18) | 1.82 (4.77) |
| Conjunctions | 1.42 (4.17) | 1.65 (3.09) | 2.43 (5.52) |
| Negations | 0.69 (3.24) | 0.45 (1.99) | 0.48 (1.92) |
| Verb | 5.34 (8.74) | 6.29 (10.68) | 7.49 (10.68) |
| Adjectives | 47.15 (23.72) | 37.66 (24.95) | 42.77 (23.13) |
| Quantity | 4.93 (9.04) | 3.38 (6.78) | 5.20 (8.50) |

## 2.3. Affect and cognition

| **LIWC category** | **Church** | **Mall** | **Park** |
| --- | --- | --- | --- |
| Cognition | 8.02 (11.64) | 11.71 (10.36) | 10.16 (10.27) |
| All-or-none terms | 0.22 (1.09) | 0.30 (1.28) | 0.24 (1.52) |
| Cognitive processes | 7.80 (11.52) | 11.42 (10.39) | 9.92 (10.11) |
| Insight | 4.50 (9.51) | 5.06 (7.49) | 5.99 (8.51) |
| Cause | 0.30 (1.19) | 0.65 (2.60) | 0.49 (2.16) |
| Discrepancy | 0.66 (2.82) | 0.62 (1.93) | 0.40 (1.66) |
| Tentative language | 0.23 (1.11) | 1.35 (4.20) | 1.11 (4.04) |
| Differentiation | 0.84 (4.35) | 1.54 (3.76) | 1.33 (5.24) |
| Memory | 0.60 (2.80) | 0.18 (1.03) | 0.30 (1.95) |
| Affect | 55.69 (31.10) | 45.61 (29.97) | 48.11 (29.46) |
| Positive tone | 33.16 (24.44) | 24.87 (27.68) | 35.65 (26.12) |
| Negative tone | 22.03 (27.83) | 20.08 (21.60) | 11.79 (16.61) |
| Emotion | 44.47 (27.66) | 33.04 (23.06) | 37.29 (25.16) |
| Positive emotion | 24.06 (20.70) | 17.25 (20.53) | 27.19 (21.16) |
| Negative emotion | 19.91 (26.60) | 15.12 (18.55) | 9.43 (16.16) |
| Anxiety | 6.74 (11.59) | 9.14 (15.98) | 2.44 (5.25) |
| Anger | 2.54 (9.90) | 1.28 (4.46) | 1.74 (5.37) |
| Sadness | 2.91 (6.91) | 0.37 (2.72) | 0.11 (0.63) |

## 2.4. Social processes and motivational drives

| **LIWC category** | **Church** | **Mall** | **Park** |
| --- | --- | --- | --- |
| Drives | 3.29 (7.57) | 1.94 (5.02) | 1.60 (4.87) |
| Affiliation | 0.59 (2.87) | 0.09 (0.68) | 0.42 (2.74) |
| Achievement | 1.22 (4.34) | 1.17 (4.12) | 1.13 (4.15) |
| Power | 1.48 (4.47) | 0.68 (3.13) | 0.05 (0.36) |
| Social | 3.98 (8.02) | 5.05 (7.19) | 1.90 (5.13) |
| Social behaviour | 3.84 (8.04) | 3.50 (6.26) | 1.61 (4.81) |
| Prosocial behaviour | 1.63 (4.85) | 0.68 (2.92) | 0.42 (2.74) |
| Politeness | 0.00 (0.00) | 0.00 (0.00) | 0.05 (0.38) |
| Conflict | 0.89 (4.17) | 0.17 (1.00) | 0.37 (2.72) |
| Morality | 0.37 (2.49) | 0.37 (2.72) | 0.00 (0.00) |
| Communication | 0.07 (0.47) | 0.39 (2.03) | 0.20 (0.90) |
| Social referents | 0.14 (0.93) | 1.24 (3.53) | 0.25 (1.11) |
| Family | 0.00 (0.00) | 0.21 (1.18) | 0.00 (0.00) |
| Friend | 0.00 (0.00) | 0.09 (0.68) | 0.10 (0.76) |

## 2.5. Physical states, needs, perception, and time

| **LIWC category** | **Church** | **Mall** | **Park** |
| --- | --- | --- | --- |
| Physical | 7.91 (13.49) | 6.95 (11.62) | 6.85 (10.11) |
| Health | 6.10 (10.63) | 2.64 (6.23) | 2.55 (5.73) |
| Illness | 0.44 (2.98) | 0.00 (0.00) | 0.00 (0.00) |
| Wellness | 0.39 (2.17) | 0.00 (0.00) | 0.00 (0.00) |
| Substances | 0.00 (0.00) | 0.00 (0.00) | 0.05 (0.38) |
| Sexual | 0.00 (0.00) | 0.11 (0.80) | 0.15 (1.13) |
| Food | 0.89 (4.17) | 2.88 (6.43) | 2.50 (6.18) |
| Death | 0.32 (2.13) | 0.00 (0.00) | 0.00 (0.00) |
| Need | 1.21 (4.62) | 2.62 (6.38) | 2.70 (6.35) |
| Want | 0.35 (1.91) | 0.89 (2.36) | 0.44 (1.89) |
| Acquire | 0.41 (2.21) | 0.27 (1.00) | 0.77 (3.37) |
| Lack | 0.98 (4.19) | 2.56 (6.22) | 2.50 (6.18) |
| Fulfill | 1.21 (4.62) | 2.01 (5.86) | 2.35 (6.41) |
| Fatigue | 5.27 (9.49) | 4.26 (8.45) | 4.72 (10.35) |
| Reward | 1.21 (4.62) | 1.43 (4.79) | 0.42 (2.74) |
| Risk | 3.23 (7.10) | 1.61 (5.74) | 1.11 (4.62) |
| Curiosity | 2.73 (8.22) | 3.73 (8.40) | 4.65 (9.01) |
| Allure | 4.95 (8.14) | 3.72 (6.85) | 6.95 (9.78) |
| Perception | 13.63 (13.57) | 15.08 (12.92) | 11.56 (13.00) |
| Attention | 2.39 (7.47) | 4.99 (10.50) | 2.21 (6.09) |
| Motion | 0.58 (2.32) | 0.74 (2.36) | 0.48 (1.44) |
| Space | 4.22 (7.43) | 3.55 (5.95) | 4.03 (7.28) |
| Visual | 0.44 (2.08) | 1.07 (3.40) | 0.49 (1.81) |
| Auditory | 2.60 (6.20) | 1.79 (4.65) | 1.65 (4.87) |
| Feeling | 3.89 (6.95) | 3.51 (7.40) | 2.92 (7.02) |
| Time | 1.77 (4.25) | 1.30 (4.17) | 2.48 (5.03) |
| Past focus | 1.20 (4.09) | 1.74 (6.57) | 1.33 (3.85) |
| Present focus | 2.01 (4.73) | 1.76 (3.64) | 3.20 (7.42) |
| Future focus | 0.85 (2.94) | 1.26 (4.41) | 1.29 (4.26) |

## 2.6. Culture, lifestyle, conversation, and punctuation

| **LIWC category** | **Church** | **Mall** | **Park** |
| --- | --- | --- | --- |
| Culture | 0.00 (0.00) | 0.13 (0.97) | 0.00 (0.00) |
| Technology | 0.00 (0.00) | 0.13 (0.97) | 0.00 (0.00) |
| Lifestyle | 3.31 (6.54) | 3.44 (6.82) | 3.02 (6.16) |
| Leisure | 2.17 (5.43) | 1.83 (5.75) | 2.76 (6.16) |
| Home | 0.67 (3.14) | 0.42 (2.34) | 0.17 (1.07) |
| Work | 0.09 (0.57) | 0.49 (1.80) | 0.08 (0.44) |
| Money | 0.00 (0.00) | 0.69 (3.33) | 0.00 (0.00) |
| Religion | 0.39 (2.17) | 0.00 (0.00) | 0.00 (0.00) |
| Conversation | 0.07 (0.47) | 0.00 (0.00) | 0.97 (3.78) |
| Netspeak | 0.00 (0.00) | 0.00 (0.00) | 0.10 (0.76) |
| Assent | 0.07 (0.47) | 0.00 (0.00) | 0.86 (3.73) |
| All punctuation | 1.93 (5.30) | 6.25 (27.76) | 2.66 (5.71) |
| Period | 0.54 (2.95) | 4.50 (27.44) | 0.34 (1.43) |
| Comma | 0.43 (1.74) | 0.37 (1.47) | 1.14 (3.09) |
| Question marks | 0.07 (0.47) | 0.00 (0.00) | 0.00 (0.00) |
| Apostrophes | 0.61 (2.71) | 0.64 (1.78) | 0.37 (1.65) |
| Other punctuation | 0.28 (1.86) | 0.74 (5.44) | 0.82 (3.96) |
