# Supplementary material for: Language-based affect assessments capture experiment-induced changes beyond rating scales
Source: Behav Res Methods. 2026 Nov 3;58(12):340. doi: 10.3758/s13428-026-03200-z (PMC13736416; doi:10.3758/s13428-026-03200-z)
Supplement: Supplementary file 2 [file 13428_2026_3200_MOESM2_ESM.docx]

# Table S13 *Supplementary confusion matrices*

Confusion matrices of results from Table 2 and table 3.

# 1. Confusion Matrices

Rows represent truth; columns represent estimate.

## 1.1. Table 2 | Best models selected by online holdout | 1. Best PANAS model | CV

| Truth | church | mall | park |
| --- | --- | --- | --- |
| church | 94 | 52 | 54 |
| mall | 60 | 94 | 42 |
| park | 58 | 33 | 99 |

## 1.2. Table 2 | Best models selected by online holdout | 1. Best PANAS model | Online holdout

| Truth | church | mall | park |
| --- | --- | --- | --- |
| church | 23 | 14 | 17 |
| mall | 11 | 26 | 15 |
| park | 14 | 4 | 29 |

## 1.3. Table 2 | Best models selected by online holdout | 1. Best PANAS model | Offline holdout

| Truth | church | mall | park |
| --- | --- | --- | --- |
| church | 14 | 19 | 12 |
| mall | 27 | 9 | 18 |
| park | 33 | 10 | 11 |

## 1.4. Table 2 | Best models selected by online holdout | 2. Best embedding model | CV

| Truth | church | mall | park |
| --- | --- | --- | --- |
| church | 89 | 56 | 55 |
| mall | 46 | 122 | 28 |
| park | 47 | 33 | 110 |

## 1.5. Table 2 | Best models selected by online holdout | 2. Best embedding model | Online holdout

| Truth | church | mall | park |
| --- | --- | --- | --- |
| church | 22 | 12 | 20 |
| mall | 13 | 33 | 6 |
| park | 11 | 6 | 30 |

## 1.6. Table 2 | Best models selected by online holdout | 2. Best embedding model | Offline holdout

| Truth | church | mall | park |
| --- | --- | --- | --- |
| church | 25 | 8 | 11 |
| mall | 22 | 26 | 6 |
| park | 16 | 15 | 22 |

## 1.7. Table 2 | Best models selected by online holdout | 3. Embeddings + PANAS | CV

| Truth | church | mall | park |
| --- | --- | --- | --- |
| church | 89 | 56 | 55 |
| mall | 46 | 122 | 28 |
| park | 47 | 33 | 110 |

## 1.8. Table 2 | Best models selected by online holdout | 3. Embeddings + PANAS | Online holdout

| Truth | church | mall | park |
| --- | --- | --- | --- |
| church | 23 | 11 | 20 |
| mall | 15 | 28 | 9 |
| park | 11 | 6 | 30 |

## 1.9. Table 2 | Best models selected by online holdout | 3. Embeddings + PANAS | Offline holdout

| Truth | church | mall | park |
| --- | --- | --- | --- |
| church | 25 | 6 | 13 |
| mall | 22 | 25 | 7 |
| park | 16 | 14 | 23 |

## 1.10. Table 2 | Best models selected by offline holdout | 1. Best PANAS model | CV

| Truth | church | mall | park |
| --- | --- | --- | --- |
| church | 182 | 2 | 16 |
| mall | 178 | 6 | 12 |
| park | 173 | 6 | 11 |

## 1.11. Table 2 | Best models selected by offline holdout | 1. Best PANAS model | Online holdout

| Truth | church | mall | park |
| --- | --- | --- | --- |
| church | 54 | 0 | 0 |
| mall | 52 | 0 | 0 |
| park | 47 | 0 | 0 |

## 1.12. Table 2 | Best models selected by offline holdout | 1. Best PANAS model | Offline holdout

| Truth | church | mall | park |
| --- | --- | --- | --- |
| church | 45 | 0 | 0 |
| mall | 54 | 0 | 0 |
| park | 54 | 0 | 0 |

## 1.13. Table 2 | Best models selected by offline holdout | 2. Best embedding model | CV

| Truth | church | mall | park |
| --- | --- | --- | --- |
| church | 60 | 74 | 66 |
| mall | 42 | 121 | 33 |
| park | 52 | 32 | 106 |

## 1.14. Table 2 | Best models selected by offline holdout | 2. Best embedding model | Online holdout

| Truth | church | mall | park |
| --- | --- | --- | --- |
| church | 21 | 13 | 20 |
| mall | 11 | 32 | 9 |
| park | 13 | 8 | 26 |

## 1.15. Table 2 | Best models selected by offline holdout | 2. Best embedding model | Offline holdout

| Truth | church | mall | park |
| --- | --- | --- | --- |
| church | 32 | 4 | 8 |
| mall | 22 | 28 | 4 |
| park | 26 | 12 | 15 |

## 1.16. Table 2 | Best models selected by offline holdout | 3. Embeddings + PANAS | CV

| Truth | church | mall | park |
| --- | --- | --- | --- |
| church | 60 | 74 | 66 |
| mall | 42 | 121 | 33 |
| park | 52 | 32 | 106 |

## 1.17. Table 2 | Best models selected by offline holdout | 3. Embeddings + PANAS | Online holdout

| Truth | church | mall | park |
| --- | --- | --- | --- |
| church | 21 | 13 | 20 |
| mall | 11 | 32 | 9 |
| park | 13 | 8 | 26 |

## 1.18. Table 2 | Best models selected by offline holdout | 3. Embeddings + PANAS | Offline holdout

| Truth | church | mall | park |
| --- | --- | --- | --- |
| church | 32 | 4 | 8 |
| mall | 23 | 27 | 4 |
| park | 26 | 12 | 15 |

## 1.19. Table 3 | PANAS | Online

| Truth | church | mall | park |
| --- | --- | --- | --- |
| church | 11 | 21 | 22 |
| mall | 8 | 22 | 22 |
| park | 9 | 7 | 31 |

## 1.20. Table 3 | PANAS | Offline

| Truth | church | mall | park |
| --- | --- | --- | --- |
| church | 9 | 19 | 17 |
| mall | 8 | 20 | 26 |
| park | 12 | 18 | 24 |

## 1.21. Table 3 | Valence | Online

| Truth | church | mall | park |
| --- | --- | --- | --- |
| church | 9 | 12 | 33 |
| mall | 8 | 26 | 18 |
| park | 3 | 8 | 36 |

## 1.22. Table 3 | Valence | Offline

| Truth | church | mall | park |
| --- | --- | --- | --- |
| church | 9 | 19 | 17 |
| mall | 9 | 31 | 14 |
| park | 9 | 11 | 34 |

## 1.23. Table 3 | Arousal | Online

| Truth | church | mall | park |
| --- | --- | --- | --- |
| church | 9 | 18 | 27 |
| mall | 10 | 29 | 13 |
| park | 14 | 13 | 20 |

## 1.24. Table 3 | Arousal | Offline

| Truth | church | mall | park |
| --- | --- | --- | --- |
| church | 11 | 18 | 16 |
| mall | 14 | 30 | 10 |
| park | 20 | 23 | 11 |

## 1.25. Table 3 | Anger | Online

| Truth | church | mall | park |
| --- | --- | --- | --- |
| church | 11 | 7 | 36 |
| mall | 9 | 20 | 23 |
| park | 6 | 6 | 35 |

## 1.26. Table 3 | Anger | Offline

| Truth | church | mall | park |
| --- | --- | --- | --- |
| church | 13 | 12 | 20 |
| mall | 11 | 27 | 16 |
| park | 10 | 14 | 30 |

## 1.27. Table 3 | Anticipate | Online

| Truth | church | mall | park |
| --- | --- | --- | --- |
| church | 54 | 0 | 0 |
| mall | 52 | 0 | 0 |
| park | 47 | 0 | 0 |

## 1.28. Table 3 | Anticipate | Offline

| Truth | church | mall | park |
| --- | --- | --- | --- |
| church | 45 | 0 | 0 |
| mall | 54 | 0 | 0 |
| park | 54 | 0 | 0 |

## 1.29. Table 3 | Disgust | Online

| Truth | church | mall | park |
| --- | --- | --- | --- |
| church | 22 | 10 | 22 |
| mall | 15 | 22 | 15 |
| park | 13 | 9 | 25 |

## 1.30. Table 3 | Disgust | Offline

| Truth | church | mall | park |
| --- | --- | --- | --- |
| church | 25 | 12 | 8 |
| mall | 25 | 23 | 6 |
| park | 23 | 16 | 15 |

## 1.31. Table 3 | Fear | Online

| Truth | church | mall | park |
| --- | --- | --- | --- |
| church | 8 | 12 | 34 |
| mall | 11 | 18 | 23 |
| park | 6 | 9 | 32 |

## 1.32. Table 3 | Fear | Offline

| Truth | church | mall | park |
| --- | --- | --- | --- |
| church | 14 | 16 | 15 |
| mall | 19 | 18 | 17 |
| park | 16 | 7 | 31 |

## 1.33. Table 3 | Joy | Online

| Truth | church | mall | park |
| --- | --- | --- | --- |
| church | 7 | 23 | 24 |
| mall | 5 | 30 | 17 |
| park | 6 | 12 | 29 |

## 1.34. Table 3 | Joy | Offline

| Truth | church | mall | park |
| --- | --- | --- | --- |
| church | 7 | 27 | 11 |
| mall | 9 | 30 | 15 |
| park | 15 | 13 | 26 |

## 1.35. Table 3 | Sadness | Online

| Truth | church | mall | park |
| --- | --- | --- | --- |
| church | 7 | 14 | 33 |
| mall | 5 | 23 | 24 |
| park | 8 | 9 | 30 |

## 1.36. Table 3 | Sadness | Offline

| Truth | church | mall | park |
| --- | --- | --- | --- |
| church | 13 | 18 | 14 |
| mall | 15 | 20 | 19 |
| park | 16 | 7 | 31 |

## 1.37. Table 3 | Surprise | Online

| Truth | church | mall | park |
| --- | --- | --- | --- |
| church | 40 | 10 | 4 |
| mall | 28 | 19 | 5 |
| park | 37 | 8 | 2 |

## 1.38. Table 3 | Surprise | Offline

| Truth | church | mall | park |
| --- | --- | --- | --- |
| church | 30 | 14 | 1 |
| mall | 25 | 27 | 2 |
| park | 32 | 22 | 0 |

## 1.39. Table 3 | Trust | Online

| Truth | church | mall | park |
| --- | --- | --- | --- |
| church | 35 | 19 | 0 |
| mall | 18 | 34 | 0 |
| park | 34 | 13 | 0 |

## 1.40. Table 3 | Trust | Offline

| Truth | church | mall | park |
| --- | --- | --- | --- |
| church | 27 | 18 | 0 |
| mall | 15 | 39 | 0 |
| park | 28 | 26 | 0 |

*Note.* Table 3: CV = Cross-validated, PANAS = Positive and Negative Affect Schedule. N_training_ = 586; N_online and offline holdout_ = 153.
Table 3: The pre-trained large language model is Mixed Bread. Best valence and arousal model: Valence and arousal trained from Warriner et al. (2013; for other valence and arousal models see Table S8). More model assessments are in SM Table S7. The basic emotions model is trained based on Mohammad & Turney (2013). No removal of any words.
