# Supplementary material for: Language-based affect assessments capture experiment-induced changes beyond rating scales
Source: Behav Res Methods. 2026 Nov 3;58(12):340. doi: 10.3758/s13428-026-03200-z (PMC13736416; doi:10.3758/s13428-026-03200-z)
Supplement: Supplementary file 3 [file 13428_2026_3200_MOESM3_ESM.docx]

**Appendix A**

**Removing condition-related contextual words**

According to the pre-registration, direct references to the experimental conditions (i.e., “Mall,” “Church,” and “Park”) were excluded from the post-induction responses used in the classification analyses. To further reduce potential contextual leakage, we explored additional, more stringent filtering procedures. These procedures comprised three successive approaches:

i) Automated identification of context-sensitive terms using ChatGPT-4o; ii) Augmentation of the automatically generated list through manual review to ensure comprehensive exclusion of context-revealing words; and iii) Application of the above filtering steps followed by restriction of the text to only affect-related terms derived from the Linguistic Inquiry and Word Count (LIWC) dictionaries for positive and negative emotion (Boyd et al., 2022).

For the automated identification step, we used ChatGPT-4o in two rounds. In each round, text data from 50 participants per condition were batched and provided to the model to identify words referencing place or environmental cues rather than affective content. The first round focused on three conditions (church, mall, park), while the second included four conditions (adding “mollan”). Both prompts instructed the model to list only the words found in the text without additional commentary.

***Prompt for Round 1:*** Participants described their emotions in different places, including a church, a shopping mall, and a park. Make a list of words that should be removed because they reference the place or environmental cues rather than describing an emotion. List only words within the text.

***Prompt for Round 2:*** Participants described their emotions in different places, including a church, a shopping mall, a park, and an open-air market. Make a list of words that should be removed because they reference the place or environmental cues rather than describing an emotion. List only words within the text below.

The final list comprised 215 unique words (presented in Table S5a) that were removed from post-intervention language-based affect descriptions in both the online and offline datasets. Table S5b reports the frequencies of words with counts greater than one and those exclusive to a single condition.
